# Review on solving the inverse problem in EEG source analysis

**DOI:** 10.1186/1743-0003-5-25

**Published:** 2008-11-07

**Authors:** Roberta Grech, Tracey Cassar, Joseph Muscat, Kenneth P Camilleri, Simon G Fabri, Michalis Zervakis, Petros Xanthopoulos, Vangelis Sakkalis, Bart Vanrumste

**Affiliations:** 1 iBERG, University of Malta, Malta; 2 Department of Systems and Control Engineering, Faculty of Engineering, University of Malta, Malta; 3 Department of Electronic and Computer Engineering, Technical University of Crete, Crete; 4 Institute of Computer Science, Foundation for Research and Technology, Heraklion 71110, Greece; 5 ESAT, KU Leuven, Belgium; 6 MOBILAB, IBW, K.H. Kempen, Geel, Belgium

## Abstract

In this primer, we give a review of the inverse problem for EEG source localization. This is intended for the researchers new in the field to get insight in the state-of-the-art techniques used to find approximate solutions of the brain sources giving rise to a scalp potential recording. Furthermore, a review of the performance results of the different techniques is provided to compare these different inverse solutions. The authors also include the results of a Monte-Carlo analysis which they performed to compare four non parametric algorithms and hence contribute to what is presently recorded in the literature. An extensive list of references to the work of other researchers is also provided.

This paper starts off with a mathematical description of the inverse problem and proceeds to discuss the two main categories of methods which were developed to solve the EEG inverse problem, mainly the non parametric and parametric methods. The main difference between the two is to whether a fixed number of dipoles is assumed a priori or not. Various techniques falling within these categories are described including minimum norm estimates and their generalizations, LORETA, sLORETA, VARETA, S-MAP, ST-MAP, Backus-Gilbert, LAURA, Shrinking LORETA FOCUSS (SLF), SSLOFO and ALF for non parametric methods and beamforming techniques, BESA, subspace techniques such as MUSIC and methods derived from it, FINES, simulated annealing and computational intelligence algorithms for parametric methods. From a review of the performance of these techniques as documented in the literature, one could conclude that in most cases the LORETA solution gives satisfactory results. In situations involving clusters of dipoles, higher resolution algorithms such as MUSIC or FINES are however preferred. Imposing reliable biophysical and psychological constraints, as done by LAURA has given superior results. The Monte-Carlo analysis performed, comparing WMN, LORETA, sLORETA and SLF, for different noise levels and different simulated source depths has shown that for single source localization, regularized sLORETA gives the best solution in terms of both localization error and ghost sources. Furthermore the computationally intensive solution given by SLF was not found to give any additional benefits under such simulated conditions.

## 1 Introduction

Over the past few decades, a variety of techniques for non-invasive measurement of brain activity have been developed, one of which is source localization using electroencephalography (EEG). It uses measurements of the voltage potential at various locations on the scalp (in the order of microvolts (*μV*)) and then applies signal processing techniques to estimate the current sources inside the brain that best fit this data.

It is well established [1] that neural activity can be modelled by currents, with activity during fits being well-approximated by current dipoles. The procedure of source localization works by first finding the scalp potentials that would result from hypothetical dipoles, or more generally from a current distribution inside the head – the forward problem; this is calculated or derived only once or several times depending on the approach used in the inverse problem and has been discussed in the corresponding review on solving the forward problem [2]. Then, in conjunction with the actual EEG data measured at specified positions of (usually less than 100) electrodes on the scalp, it can be used to work back and estimate the sources that fit these measurements – the inverse problem. The accuracy with which a source can be located is affected by a number of factors including head-modelling errors, source-modelling errors and EEG noise (instrumental or biological) [3]. The standard adopted by Baillet *et. al*. in [4] is that spatial and temporal accuracy should be at least better than 5 mm and 5 ms, respectively.

In this primer, we give a review of the inverse problem in EEG source localization. It is intended for the researcher who is new in the field to get insight in the state-of-the-art techniques used to get approximate solutions. It also provides an extensive list of references to the work of other researchers. The primer starts with a mathematical formulation of the problem. Then in Section 3 we proceed to discuss the two main categories of inverse methods: non parametric methods and parametric methods. For the first category we discuss minimum norm estimates and their generalizations, the Backus-Gilbert method, Weighted Resolution Optimization, LAURA, shrinking and multiresolution methods. For the second category, we discuss the non-linear least-squares problem, beamforming approaches, the Multiple-signal Classification Algorithm (MUSIC), the Brain Electric Source Analysis (BESA), subspace techniques, simulated annealing and finite elements, and computational intelligence algorithms, in particular neural networks and genetic algorithms. In Section 4 we then give an overview of source localization errors and a review of the performance analysis of the techniques discussed in the previous section. This is then followed by a discussion and conclusion which are given in Section 5.

## 2 Mathematical formulation

**Figure 1:**
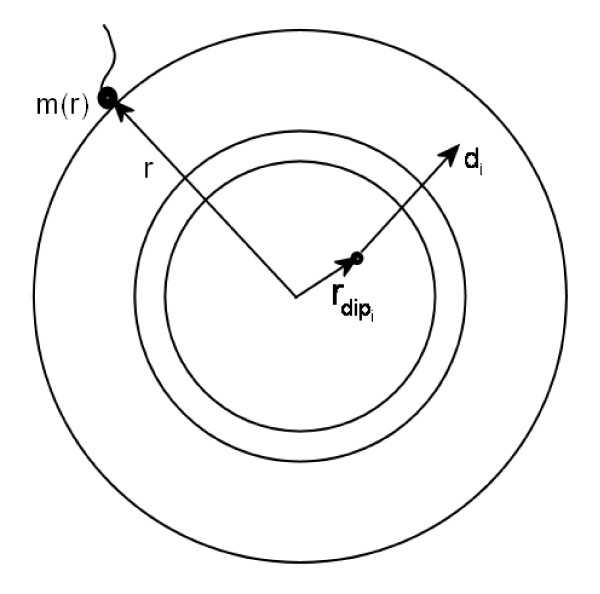
A three layer head model.

In symbolic terms, the EEG forward problem is that of finding, in a reasonable time, the potential *g*(**r**, **r**_*dip*_, **d**) at an electrode positioned on the scalp at a point having position vector **r **due to a single dipole with dipole moment **d **= *d***e**_**d **_(with magnitude *d *and orientation **e**_**d**_), positioned at **r**_*dip *_(see Figure 1). This amounts to solving Poisson's equation to find the potentials *V *on the scalp for different configurations of **r**_*dip *_and **d**. For multiple dipole sources, the electrode potential would be $m(r)=\sum_{i}g(r,r_{dip_{i}},d_{i})$. Assuming the principle of superposition, this can be rewritten as $\sum_{i}g(r,r_{dip_{i}})(d_{ix},d_{iy},d_{iz}{)}^{T}=\sum_{i}g(r,r_{dip_{i}})d_{i}e_{i}$, where **g**(**r**, $r_{dip_{i}}$) now has three components corresponding to the Cartesian *x*, *y*, *z *directions, **d**_*i *_= (*d*_*ix*_, *d*_*iy*_, *d*_*iz*_) is a vector consisting of the three dipole magnitude components, '^*T*^' denotes the transpose of a vector, *d*_*i *_= ||**d**_*i*_|| is the dipole magnitude and $e_{i}=\frac{d_{i}}{\left\|d_{i}\right\|}$ is the dipole orientation. In practice, one calculates a potential between an electrode and a reference (which can be another electrode or an average reference).

For *N *electrodes and *p *dipoles:

(1) $$m=\left[\begin{array}{c} m(r_{1})\\ \vdots \\ m(r_{N}) \end{array}\right]=\left[\begin{array}{ccc} g(r_{1},r_{dip_{1}}) & \cdots & g(r_{1},r_{dip_{p}})\\ \vdots & \ddots & \vdots \\ g(r_{N},r_{dip_{1}}) & \cdots & g(r_{N},r_{dip_{p}}) \end{array}\right]\left[\begin{array}{c} d_{1}e_{1}\\ \vdots \\ d_{p}e_{p} \end{array}\right]$$

where *i *= 1, ..., *p *and *j *= 1, ..., *N*. Each row of the gain matrix **G **is often referred to as the lead-field and it describes the current flow for a given electrode through each dipole position [5].

For *N *electrodes, *p *dipoles and *T *discrete time samples:

(2a) $$M=\left[\begin{array}{ccc} m(r_{1},1) & \cdots & m(r_{1},T)\\ \vdots & \ddots & \vdots \\ m(r_{N},1) & \cdots & m(r_{N},T) \end{array}\right]=G(\{r_{j},r_{dip_{i}}\})\left[\begin{array}{ccc} d_{1,1}e_{1} & \cdots & d_{1,T}e_{1}\\ \vdots & \ddots & \vdots \\ d_{p,1}e_{p} & \cdots & d_{p,T}e_{p} \end{array}\right]$$

(2b) $$=G(\{r_{j},r_{dip_{i}}\})D$$

where **M **is the matrix of data measurements at different times *m*(*r*, *t*) and **D **is the matrix of dipole moments at different time instants.

In the formulation above it was assumed that both the magnitude and orientation of the dipoles are unknown. However, based on the fact that apical dendrites producing the measured field are oriented normal to the surface [6], dipoles are often constrained to have such an orientation. In this case only the magnitude of the dipoles will vary and the formulation in (2a) can therefore be re-written as:

(3a) $$M=\left[\begin{array}{ccc} g(r_{1},r_{dip_{1}})e_{1} & \cdots & g(r_{1},r_{dip_{p}})e_{p}\\ \vdots & \ddots & \vdots \\ g(r_{N},r_{dip_{1}})e_{1} & \cdots & g(r_{N},r_{dip_{p}})e_{p} \end{array}\right]\left[\begin{array}{c} d_{1}\\ \vdots \\ d_{p} \end{array}\right]$$

(3b) $$=G(\{r_{j},r_{dip_{i}},e_{i}\})\left[\begin{array}{ccc} d_{1,1} & \cdots & d_{1,T}\\ \vdots & \ddots & \vdots \\ d_{p,1} & \cdots & d_{p,T} \end{array}\right]$$

(3c) $$=G(\{r_{j},r_{dip_{i}},e_{i}\})D$$

where **D **is now a matrix of dipole magnitudes at different time instants. This formulation is less underdetermined than that in the previous structure.

Generally a noise or perturbation matrix n is added to the system such that the recorded data matrix **M **is composed of:

(4) **M **= **GD + n**.

Under this notation, the inverse problem then consists of finding an estimate $\hat{D}$ of the dipole magnitude matrix given the electrode positions and scalp readings **M **and using the gain matrix **G **calculated in the forward problem. In what follows, unless otherwise stated, *T *= 1 without loss of generality.

## 3 Inverse solutions

The EEG inverse problem is an ill-posed problem because for all admissible output voltages, the solution is non-unique (since *p *>> *N*) and unstable (the solution is highly sensitive to small changes in the noisy data). There are various methods to remedy the situation (see e.g. [7-9]). As regards the EEG inverse problem, there are six parameters that specify a dipole: three spatial coordinates (*x*, *y*, *z*) and three dipole moment components (orientation angles (*θ*, *φ*) and strength *d*), but these may be reduced if some constraints are placed on the source, as described below.

Various mathematical models are possible depending on the number of dipoles assumed in the model and whether one or more of dipole position(s), magnitude(s) and orientation(s) is/are kept fixed and which, if any, of these are assumed to be known. In the literature [10] one can find the following models: a single dipole with time-varying unknown position, orientation and magnitude; a fixed number of dipoles with fixed unknown positions and orientations but varying amplitudes; fixed known dipole positions and varying orientations and amplitudes; variable number of dipoles (i.e. a dipole at each grid point) but with a set of constraints. As regards dipole moment constraints, which may be necessary to limit the search space for meaningful dipole sources, Rodriguez-Rivera *et al*. [11] discuss four dipole models with different dipole moment constraints. These are (i) constant unknown dipole moment; (ii) fixed known dipole moment orientation and variable moment magnitude; (iii) fixed unknown dipole moment orientation, variable moment magnitude; (iv) variable dipole moment orientation and magnitude.

There are two main approaches to the inverse solution: non-parametric and parametric methods. Non-parametric optimization methods are also referred to as Distributed Source Models, Distributed Inverse Solutions (DIS) or Imaging methods. In these models several dipole sources with fixed locations and possibly fixed orientations are distributed in the whole brain volume or cortical surface. As it is assumed that sources are intracellular currents in the dendritic trunks of the cortical pyramidal neurons, which are normally oriented to the cortical surface [6], fixed orientation dipoles are generally set to be normally aligned. The amplitudes (and direction) of these dipole sources are then estimated. Since the dipole location is not estimated the problem is a linear one. This means that in Equation 4, {$r_{dip_{i}}$} and possibly **e**_*i *_are determined beforehand, yielding large *p *>> *N *which makes the problem underdetermined. On the other hand, in the parametric approach few dipoles are assumed in the model whose location and orientation are unknown. Equation (4) is solved for **D**, {$r_{dip_{i}}$} and **e**_*i*_, given **M **and what is known of **G**. This is a non-linear problem due to parameters {$r_{dip_{i}}$}, **e**_*i *_appearing non-linearly in the equation.

These two approaches will now be discussed in more detail.

### 3.1 Non parametric optimization methods

Besides the Bayesian formulation explained below, there are other approaches for deriving the linear inverse operators which will be described, such as minimization of expected error and generalized Wiener filtering. Details are given in [12]. Bayesian methods can also be used to estimate a probability distribution of solutions rather than a single 'best' solution [13].

#### 3.1.1 The Bayesian framework

In general, this technique consists in finding an estimator $\hat{x}$ of **x **that maximizes the posterior distribution of **x **given the measurements **y **[4,12-15]. This estimator can be written as

$$\hat{x}=\underset{x}{\max}[p(x|y)]$$

where *p*(**x **| **y**) denotes the conditional probability density of **x **given the measurements **y**. This estimator is the most probable one with regards to measurements and *a priori *considerations.

According to Bayes' law,

$$p(x|y)=\frac{p(y|x)p(x)}{p(y)}.$$

##### The Gaussian or Normal density function

Assuming the posterior density to have a Gaussian distribution, we find

$$p(x|y)=\frac{p(x)p(y|x)}{p(y)}=\frac{\exp[-F_{\alpha }(x)]/z}{p(y)}$$

where *z *is a normalization constant called the partition function, *F*_*α*_(**x**) = *U*_1_(**x**) + *αL*(**x**) where *U*_1_(**x**) and *L*(**x**) are energy functions associated with *p*(**y **| **x**) and *p*(**x**) respectively, and *α *(a positive scalar) is a tuning or regularization parameter. Then

$$\hat{x}=\underset{x}{\min}(F_{\alpha }(x)).$$

If measurement noise is assumed to be white, Gaussian and zero-mean, one can write *U*_1_(**x**) as

*U*_1_(**x**) = ||**Kx **-
                           **y**||^2^

where **K **is a compact linear operator [7,16] (representing the forward solution) and ||.|| is the usual *L*_2 _norm. *L*(**x**) may be written as *U*_*s*_(**x**) + *U*_*t*_(**x**) where *U*_*s*_(**x**) introduces spatial (anatomical) priors and *U*_*t*_(**x**) temporal ones [4,15]. Combining the data attachment term with the prior term,

$$\hat{x}=\underset{x}{\min}(F_{\alpha }(x))=\underset{x}{\min}(||Kx-y|{|}^{2}+\alpha L(x)).$$

This equation reflects a trade off between fidelity to the data and spatial/temporal smoothness depending on the *α*.

In the above, *p*(**y **| **x**) ∝ exp(-**X**^*T*^.**X**) where **X **= **Kx **- **y**. More generally, *p*(**y **| **x**) ∝ exp(-*Tr*(**X**^*T*^.*σ*^-1^.**X**)), where *σ*^-1 ^is the data covariance matrix and '*Tr*' denotes the trace of a matrix.

##### The general Normal density function

Even more generally, *p*(**y **| **x**) ∝ exp(-*Tr*((**X **- *μ*)^*T*^.*σ*^-1^.(**X **- *μ*))), where *μ *is the mean value of **X**. Suppose **R **is the variance-covariance matrix when a Gaussian noise component is assumed and **Y **is the matrix corresponding to the measurements **y**. The R-norm is defined as follows:

$$\left||Y-KX|{|}_{R}^{2}=Tr[{\left(Y-KX\right)}^{T}R^{-1}(Y-KX)\right]$$

##### Non-Gaussian priors

Non-Gaussian priors include entropy metrics and *L*_*p *_norms with *p *< 2 i.e. *L*(**x**) = ||**x**||_*p*_.

Entropy is a probabilistic concept appearing in information theory and statistical mechanics. Assuming **x **∈ ℝ^*n *^consists of positive entries *x*_*i *_> 0, *i *= 1, ..., *n *the entropy is defined as

$$ℰ(x)=-\sum_{i=1}^{n}x_{i}\log\left(\frac{x_{i}}{x_{i}^{*}}\right)$$

where $x_{i}^{*}$ > 0 is a is a given constant. The information contained in **x **relative to $x_{i}^{*}$ is the negative of the entropy. If it is required to find **x **such that only the data **Kx **= **y **is used, the information subject to the data needs to be minimized, that is, the entropy has to be maximized. The mathematical justification for the choice *L*(**x**) = -ℰ(**x**) is that it yields the solution which is most 'objective' with respect to missing information. The maximum entropy method has been used with success in image restoration problems where prominent features from noisy data are to be determined.

As regards *L*_*p *_norms with *p *< 2, we start by defining these norms. For a matrix **A**, $||A|{|}_{p}=\sqrt{\sum_{i,j}|a_{ij}{|}^{p}p}$ where *a*_*ij *_are the elements of **A**. The defining feature of these prior models is that they are concentrated on images with low average amplitude with few outliers standing out. Thus, they are suitable when the prior information is that the image contains small and well localized objects as, for example, in the localization of cortical activity by electric measurements.

As *p *is reduced the solutions will become increasingly sparse. When *p *= 1 [17] the problem can be modified slightly to be recast as a linear program which can be solved by a simplex method. In this case it is the sum of the absolute values of the solution components that is minimized. Although the solutions obtained with this norm are sparser than those obtained with the *L*_2 _norm, the orientation results were found to be less clear [17]. Another difference is that while the localization results improve if the number of electrodes is increased in the case of the *L*_2 _approach, this is not the case with the *L*_1 _approach which requires an increase in the number of grid points for correct localization. A third difference is that while both approaches perform badly in the presence of noisy data, the *L*_1 _approach performs even worse than the *L*_2 _approach. For *p *< 1 it is possible to show that there exists a value 0 <*p *< 1 for which the solution is maximally sparse. The non-quadratic formulation of the priors may be linked to previous works using Markov Random Fields [18,19]. Experiments in [20] show that the *L*_1 _approach demands more computational effort in comparision with *L*_2 _approaches. It also produced some spurious sources and the source distribution of the solution was very different from the simulated distribution.

##### Regularization methods

Regularization is the approximation of an ill-posed problem by a family of neighbouring well-posed problems. There are various regularization methods found in the literature depending on the choice of *L*(**x**). The aim is to find the best-approximate solution **x**^*δ *^of **Kx **= **y **in the situation that the 'noiseless data' **y **are not known precisely but that only a noisy representation **y**^*δ *^with ||**y**^*δ *^- **y**|| ≤ *δ *is available. Typically **y**^*δ *^would be the real (noisy) signal. In general, an $x_{\alpha }^{\delta }$ is found which minimizes

*F*_*α*_(**x**) = ||**Kx **- **y**^*δ*^||^2 ^+ *αL*(**x**).

In Tikhonov regularization, *L*(**x**) = ||**x**||^2 ^so that an $x_{\alpha }^{\delta }$ is found which minimizes

*F*_*α*_(**x**) = ||**Kx **- **y**^*δ*^||^2 ^+
                     *α*||**x**||^2^.

It can be shown (in Appendix) that

$$x_{\alpha (\delta )}^{\delta }={\left(K^{*}K+\alpha I\right)}^{-1}K^{*}y^{\delta }$$

where K* is the adjoint of **K**. Since (K*K + *α***I**)^-1^K* = K*(KK* + *α***I**)^-1 ^(proof in Appendix),

$$x_{\alpha (\delta )}^{\delta }=K^{*}{\text{(}KK^{*}+\alpha I)}^{-1}y^{\delta }.$$

Another choice of *L*(**x**) is

(5) *L*(**x**) =
                     ||**Ax**||^2^

where **A **is a linear operator. The minimum is obtained when

(6) $$x_{\alpha (\delta )}^{\delta }={\left(K^{*}K+\alpha A^{*}A\right)}^{-1}K^{*}y$$

In particular, if **A **= **∇ **where **∇ **is the gradient operator, then $x_{\alpha (\delta )}^{\delta }$ = (K*K + *α*∇^*T*^∇)^-1^K*y. If **A **= **ΔB**, where **Δ **is the Laplacian operator, then $x_{\alpha (\delta )}^{\delta }$ = (K*K + *α*B*Δ^*T*^Δ**B**)^-1^K*y.

The regularization parameter *α *must find a good compromise between the residual norm ||**Kx **- **y**^*δ*^|| and the norm of the solution ||**Ax**||. In other words it must find a balance between the perturbation error in **y **and the regularization error in the regularized solution.

Various methods [7-9] exist to estimate the optimal regularization parameter and these fall mainly in two categories:

1. Those based on a good estimate of ||*ϵ*|| where *ϵ *is the noise in the measured vector **y**^*δ*^.

2. Those that do not require an estimate of ||*ϵ*||.

The discrepancy principle is the main method based on ||*ϵ*||. In effect it chooses *α *such that the residual norm for the regularized solution satisfies the following condition:

||**Kx **- **y**^*δ*^|| = ||*ϵ*||

As expected, failure to obtain a good estimate of *ϵ *will yield a value for *α *which is not optimal for the expected solution.

Various other methods of estimating the regularization parameter exist and these fall mainly within the second category. These include, amongst others, the

1. **L**-curve method

2. General-Cross Validation method

3. Composite Residual and Smoothing Operator (CRESO)

4. Minimal Product method

5. Zero crossing

**Figure 2:**
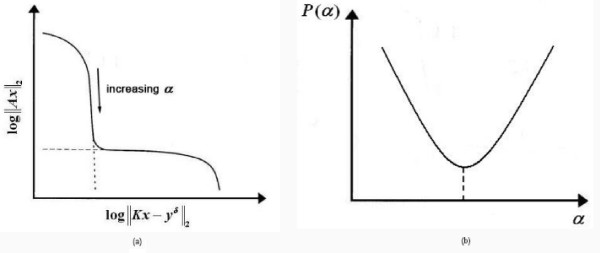
**Methods to estimate the regularization parameter**. (a) L-curve (b) Minimal Product Curve.

The **L**-curve method [21-23] provides a log-log plot of the semi-norm ||**Ax**|| of the regularized solution against the corresponding residual norm ||**Kx **- **y**^*δ*^|| (Figure 2a). The resulting curve has the shape of an 'L', hence its name, and it clearly displays the compromise between minimizing these two quantities. Thus, the best choice of alpha is that corresponding to the corner of the curve. When the regularization method is continuous, as is the case in Tikhonov regularization, the **L**-curve is a continuous curve. When, however, the regularization method is discrete, the **L**-curve is also discrete and is then typically represented by a spline curve in order to find the corner of the curve.

Similar to the **L**-curve method, the Minimal Product method [24] aims at minimizing the upper bound of the solution and the residual simultaneously (Figure 2b). In this case the optimum regularization parameter is that corresponding to the minimum value of function *P *which gives the product between the norm of the solution and the norm of the residual. This approach can be adopted to both continuous and discrete regularization.

*P*(*α*) =
                           ||**Ax**(*α*)||.||**Kx**(*α*) -
                           **y**^*δ*^||

Another well known regularization method is the Generalized Cross Validation (GCV) method [21,25] which is based on the assumption that **y **is affected by normally distributed noise. The optimum alpha for GCV is that corresponding to the minimum value for the function *G*:

$$G=\frac{||Kx(\alpha )-y^{\delta }|{|}^{2}}{{\left(Tr(I-KT)\right)}^{2}}$$

where **T **is the inverse operator of matrix **K**. Hence the numerator measures the discrepancy between the estimated and measured signal **y**^*δ *^while the denominator measures the discrepancy of matrix KT from the identity matrix.

The regularization parameter as estimated by the Composite Residual and Smoothing Operator (CRESO) [23,24] is that which maximizes the derivative of the difference between the residual norm and the semi-norm i.e. the derivative of *B*(*α*):

(7) *B*(*α*) =
                           *α*^2^||**Ax**(*α*)||^2
                        ^- ||**Kx**(*α*) - **y**^*δ*^||^2^

Unlike the other described methods for finding the regularization parameter, this method works only for continuous regularization such as Tikhonov.

The final approach to be discussed here is the zero-crossing method [23] which finds the optimum regularization parameter by solving *B*(*α*) = 0 where *B *is as defined in Equation (7). Thus the zero-crossing is basically another way of obtaining the **L**-curve corner.

One must note that the above estimators for $x_{\alpha (\delta )}^{\delta }$ are the same as those that result from the minimization of ||**Ax**|| subject to **Kx **= **y**. In this case **x **= **K**^(*)^(**KK**^(*)^)^-1^**y **where **K**^(*) ^= (AA*)^-1^K* is found with respect to the inner product ⟨⟨**x**, **y**⟩⟩ = ⟨**Ax**, **Ay**⟩. This leads to the estimator,

**x **=
                           (A*A)^-1^K*(**K**(AA*)^-1^K*)^-1^**y**

which, if regularized, can be shown to be equivalent to (6).

As regards the EEG inverse problem, using the notation used in the description of the forward problem in Section ??, the Bayesian methods find an estimate $\hat{D}$ of **D **such that

$$\hat{D}=\min(U(D))$$

where

$$U(D)=||M-GD|{|}_{R}^{2}+\alpha L(D).$$

As an example, in [26] one finds that the linear operator **A **in Equation (5) is taken to be a matrix **A **whose rows represent the averages (linear combinations) of the true sources. One choice of the matrix **A **is given by

$$\begin{array}{lll} A_{ij} & = & A_{3(p-1)+k,3(q-1)+m}\\ & = & \begin{array}{cccc} w_{j}\exp^{-d_{pq}^{2}/\sigma_{i}^{2}} & \text{for} & k=m & \text{and zero otherwise} \end{array} \end{array}$$

In the above equation, the subscripts *p*, *q *are used to indicate grid points in the volume representing the brain and the subscripts *k*, *m *are used to represent Cartesian coordinates *x*, *y *and *z *(i.e. they take values 1,2,3), *d*_*pq *_represents the Euclidean distances between the *p*th and *q*th grid points. The coefficients *w*_*j *_can be used to describe a column scaling by a diagonal matrix while *σ*_*i *_controls the spatial resolution. In particular, if *σ*_*i *_→ 0 and *w*_*j *_= 1 the minimum norm solution described below is obtained.

In the next subsections we review some of the most common choices for *L*(**D**).

##### Minimum norm estimates (MNE)

Minimum norm estimates [5,27,28] are based on a search for the solution with minimum power and correspond to Tikhonov regularization. This kind of estimate is well suited to distributed source models where the dipole activity is likely to extend over some areas of the cortical surface.

*L*(**D**) =
                     ||**D**||^2^

$${\hat{D}}_{MNE}={\left(G^{T}G+\alpha I_{p}\right)}^{-1}G^{T}M$$

or

$${\hat{D}}_{MNE}=G^{T}{\left(GG^{T}+\alpha I_{N}\right)}^{-1}M$$

The first equation is more suitable when *N *> *p *while the second equation is more suitable when *p *> *N*. If we let **T**_*MNE *_be the inverse operator **G**^*T*^(**GG**^*T *^+ *α***I**_*N*_)^-1^, then **T**_*MNE*_**G **is called the resolution matrix and this would ideally the identity matrix. It is claimed [5,27] that MNEs produce very poor estimation of the true source locations with both the realistic and sphere models.

A more general minimum-norm inverse solution assumes that both the noise vector n and the dipole strength **D **are normally distributed with zero mean and their covariance matrices are proportional to the identity matrix and are denoted by **C **and **R **respectively. The inverse solution is given in [14]:

$${\hat{D}}_{MNE}=RG^{T}{\left(GRG^{T}+C\right)}^{-1}M$$

**R**_*ij *_can also be taken to be equal to *σ*_*i*_*σ*_*j*_*Corr*(*i*, *j*) where $\sigma_{i}^{2}$ is the variance of the strength of the *i*th dipole and *Corr*(*i*, *j*) is the correlation between the strengths of the *i*th and *j*th dipoles. Thus any *a priori *information about correlation between the dipole strengths at different locations can be used as a constraint. **R **can also be taken as $\sqrt{R_{ii}R_{jj}}(Corr(i,j))$ where $R_{ii}=f\left(\frac{1}{\zeta_{i}}\right)$ is such that it is large when the measure *ζ*_*i *_of projection onto the noise subspace is small. The matrix **C **can be taken as *σ*^2^I if it is assumed that the sensor noise is additive and white with constant variance *σ*^2^. **R **can also be constructed in such a way that it is equal to **UU**^*T *^where **U **is an orthonormal set of arbitrary basis vectors [12]. The new inverse operator using these arbitrary basis functions is the original forward solution projected onto the new basis functions.

##### Weighted minimum norm estimates (WMNE)

The Weighted Minimum Norm algorithm compensates for the tendency of MNEs to favour weak and surface sources. This is done by introducing a 3*p *× 3*p *weighting matrix **W**:

(8) $$\begin{array}{c} L(D)=||WD|{|}^{2}\\ {\hat{D}}_{WMNE}={\left(G^{T}G+\alpha W^{T}W\right)}^{-1}G^{T}M \end{array}$$

or

$${\hat{D}}_{WMNE}={\left(W^{T}W\right)}^{-1}G^{T}{\left(G{\left(W^{T}W\right)}^{-1}G^{T}+\alpha I_{N}\right)}^{-1}M$$

**W **can have different forms but the simplest one is based on the norm of the columns of the matrix **G**: **W **= **Ω **⊗ **I**_3_, where ⊗ denotes the Kronecker product and **Ω **is a diagonal *p *× *p *matrix with $\Omega_{\beta \beta }=\sqrt{\sum_{\alpha =1}^{N}g(r_{\alpha },r_{dip_{\beta }})\cdot g{\left(r_{\alpha },r_{dip_{\beta }}\right)}^{T}}$, for *β *= 1, ..., *p*.

##### MNE with FOCUSS (Focal underdetermined system solution)

This is a recursive procedure of weighted minimum norm estimations, developed to give some focal resolution to linear estimators on distributed source models [5,27,29,30]. Weighting of the columns of **G **is based on the mag nitudes of the sources of the previous iteration. The Weighted Minimum Norm compensates for the lower gains of deeper sources by using lead-field normalization.

(9) $${\hat{D}}_{FOCUSS|i}=W_{i}W_{i}^{T}G^{T}{\left(GW_{i}W_{i}^{T}G^{T}+\alpha I_{N}\right)}^{-1}M$$

where *i *is the index of the iteration and **W**_*i *_is a diagonal matrix computed using

(10) $$W_{i}=w_{i}W_{i-1}\text{ diag }({\hat{D}}_{i-1})$$

$w_{i}=\text{diag}\left(\frac{1}{\left|G(:,j)|\right|}\right)$, *j *∈ [1, 2, ..., *p*] is a diagonal matrix for deeper source compensation. G(:, *j*) is the *j*th column of **G**. The algorithm is initialized with the minimum norm solution ${\hat{D}}_{MNE}$, that is, $W_{0}=\text{diag}({\hat{D}}_{MNE})=\text{diag}({\hat{D}}_{0}(1),{\hat{D}}_{0}(2),...,{\hat{D}}_{0}(3p))$, where ${\hat{D}}_{0}$(*n*) represents the *n*th element of vector ${\hat{D}}_{0}$. If continued long enough, FOCUSS converges to a set of concentrated solutions equal in number to the number of electrodes.

The localization accuracy is claimed to be impressively improved in comparison to MNE. However, localization of deeper sources cannot be properly estimated. In addition to Minimum Norm, FOCUSS has also been used in conjunction with LORETA [31] as discussed below.

##### Low resolution electrical tomography (LORETA)

LORETA [5,27] combines the lead-field normalization with the Laplacian operator, thus, gives the depth-compensated inverse solution under the constraint of smoothly distributed sources. It is based on the maximum smoothness of the solution. It normalizes the columns of **G **to give all sources (close to the surface and deeper ones) the same opportunity of being reconstructed. This is better than minimum-norm methods in which deeper sources cannot be recovered because dipoles located at the surface of the source space with smaller magnitudes are priveleged. In LORETA, sources are distributed in the whole inner head volume. In this case, *L*(**D**) = ||**ΔB.D**||^2 ^and **B **= **Ω **⊗ **I**_3 _is a diagonal matrix for the column normalization of **G**.

$${\hat{D}}_{LOR}={\left(G^{T}G+\alpha B\Delta^{T}\Delta B\right)}^{-1}G^{T}M$$

or

$${\hat{D}}_{LOR}={\left(B\Delta^{T}\Delta B\right)}^{-1}G^{T}{\left(G{\left(B\Delta^{T}\Delta B\right)}^{-1}G^{T}+\alpha I_{N}\right)}^{-1}M$$

Experiments using LORETA [27] showed that some spurious activity was likely to appear and that this technique was not well suited for focal source estimation.

##### LORETA with FOCUSS [31]

This approach is similar to MNE with FOCUSS but based on LORETA rather than MNE. It is a combination of LORETA and FOCUSS, according to the following steps:

1. The current density is computed using LORETA to get ${\hat{D}}_{LOR}$.

2. The weighting matrix **W **is constructed using (10), the initial matrix being given by $W_{0}=\text{diag}({\hat{D}}_{LOR})=\text{diag}({\hat{D}}_{0}(1),{\hat{D}}_{0}(2),...,{\hat{D}}_{0}(3p))$, where ${\hat{D}}_{0}$(*n*) represents the *n*th element of vector ${\hat{D}}_{0}$.

3. The current density ${\hat{D}}_{i}$ is computed using (9).

4. Steps (2) and (3) are repeated until convergence.

##### Standardized low resolution brain electromagnetic tomography

Standardized low resolution brain electromagnetic tomography (sLORETA) [32] sounds like a modification of LORETA but the concept is quite different and it does not use the Laplacian operator. It is a method in which localization is based on images of standardized current density. It uses the current density estimate given by the minimum norm estimate ${\hat{D}}_{MNE}$ and standardizes it by using its variance, which is hypothesized to be due to the actual source variance **S**_**D **_= **I**_3*p*_, and variation due to noisy measurements $S_{M}^{noise}$ = *α***I**_*N*_. The electrical potential variance is **S**_**M **_= **GS**_**D**_**G**^*T *^+ $S_{M}^{noise}$ and the variance of the estimated current density is $S_{\hat{D}}=T_{MNE}S_{M}T_{MNE}^{T}=G^{T}{\left[GG^{T}+\alpha I_{N}\right]}^{-1}G$. This is equivalent to the resolution matrix **T**_*MNE*_**G**. For the case of EEG with unknown current density vector, sLORETA gives the following estimate of standardized current density power:

(11) $${\hat{D}}_{MNE,l}^{T}{\left\{{\left[S_{\hat{D}}\right]}_{ll}\right\}}^{-1}{\hat{D}}_{MNE,l}$$

where ${\hat{D}}_{MNE,l}$ ∈ ℝ^3 × 1 ^is the current density estimate at the *l*th voxel given by the minimum norm estimate and [$S_{\hat{D}}$]_*ll *_∈ ℝ^3 × 3 ^is the *l*th diagonal block of the resolution matrix $S_{\hat{D}}$. It was found [32] that in all noise free simulations, although the image was blurred, sLORETA had exact, zero error localization when reconstructing single sources, that is, the maximum of the current density power estimate coincided with the exact dipole location. In all noisy simulations, it had the lowest localization errors when compared with the minimum norm solution and the Dale method [33]. The Dale method is similar to the sLORETA method in that the current density estimate given by the minimum norm solution is used and source localization is based on standardized values of the current density estimates. However, the variance of the current density estimate is based only on the measurement noise, in contrast to sLORETA, which takes into account the actual source variance as well.

##### Variable resolution electrical tomography (VARETA)

VARETA [34] is a weighted minimum norm solution in which the regularization parameter varies spatially at each point of the solution grid. At points at which the regularization parameter is small, the source is treated as concentrated When the regularization parameter is large the source is estimated to be zero.

$${\hat{D}}_{VAR}=\arg\underset{D,\Lambda }{\min}(||M-GD|{|}^{2}+||\Lambda L_{3}.W.D|{|}^{2}+\tau^{2}||L.\ln(\Lambda )-\alpha |{|}^{2})$$

where **L **is a nonsingular univariate discrete Laplacian, **L**_3 _= **L **⊗ **I**_3 × 3_, where ⊗ denotes the Kronecker product, **W **is a certain weight matrix defined in the weighted minimum norm solution, **Λ **is a diagonal matrix of regularizing parameters, and parameters *τ *and *α *are introduced. *τ *controls the amount of smoothness and *α *the relative importance of each grid point. Estimators are calculated iteratively, starting with a given initial estimate **D**_0 _(which may be taken to be ${\hat{D}}_{LOR}$), **Λ**_*i *_is estimated from **D**_*i *- 1_, then **D**_*i *_from **Λ**_*i *_until one of them converges.

Simulations carried out with VARETA indicate the necessity of very fine grid spacing [34].

##### Quadratic regularization and spatial regularization (S-MAP) using dipole intensity gradients

In Quadratic regularization using dipole intensity gradients [4], *L*(**D**) = ||**∇D**||^2 ^which results in a source estimator given by

$${\hat{D}}_{QR}={\left(G^{T}G+\alpha \nabla^{T}\nabla \right)}^{-1}G^{T}M$$

or

$${\hat{D}}_{QR}={\left(\nabla^{T}\nabla \right)}^{-1}G^{T}(G{\left(\nabla^{T}\nabla \right)}^{-1})G^{T}+\alpha I_{N}{)}^{-1}M$$

The use of dipole intensity gradients gives rise to smooth variations in the solution.

Spatial regularization is a modification of Quadratic regularization. It is an inversion procedure based on a non-quadratic choice for *L*(**D**) which makes the estimator become non-linear and more suitable to detect intensity jumps [27].

$$L(D)=\sum_{n=1}^{N_{v}}\Phi_{v}(\nabla D_{|v})$$

where *N*_*v *_= *p *× *N*_*n *_and *N*_*n *_is the number of neighbours for each source *j*, **∇D**_|*v *_is the *v*th element of the gradient vector and $\Phi_{v}(u)=u^{2}/\left[1+{\left(\frac{u}{K_{v}}\right)}^{2}\right]$. *K*_*v *_= *α*_*v *_× *β*_*v *_where *α*_*v *_depends on the distance between a source and its current neighbour and *β*_*v *_depends on the discrepancy regarding orientations of two sources considered. For small gradients the local cost is quadratic, thus producing areas with smooth spatial changes in intensity, whereas for higher gradients, the associated cost is finite: **Φ**_*v*_(*u*) ≈ $K_{v}^{2}$, thus allowing the preservation of discontinuities. The estimator at the *i*th iteration is of the form

$${\hat{D}}_{i}=\Theta (G,L({\hat{D}}_{i-1}))M$$

where **Θ **is a *p *by *N *matrix depending on **G **and priors computed from the previous source estimate ${\hat{D}}_{i-1}$.

##### Spatio-temporal regularization (ST-MAP)

Time is taken into account in this model whereby the assumption is made that dipole magnitudes are evolving slowly with regard to the sampling frequency [4,15]. For a measurement taken at time *t*, assuming that ${\hat{D}}_{t-1}$ and ${\hat{D}}_{t}$ may be very close to each other means that the orthogonal projection of ${\hat{D}}_{t}$ on the hyperplane $E_{{\hat{D}}_{t-1}}^{⊥}$ perpendicular to ${\hat{D}}_{t-1}$ is 'small'. The following nonlinear equation is obtained:

$$G^{T}G+\alpha (\Delta^{t}+\beta P_{t-1}^{{⊥}^{T}}P_{t-1}^{⊥})D_{t}=G^{T}M_{t}$$

where

$$\Delta^{t}=-\nabla_{x}^{T}B_{x}^{t}\nabla_{x}^{T}\nabla_{x}-\nabla_{y}^{T}B_{y}^{t}\nabla_{y}$$

is a weighted Laplacian and

$$B_{x}^{t}=\text{diag}.{\left[b_{x}^{t}{|}_{k}\right]}_{k=1,...,p}$$

with

$$b_{x}^{t}{|}_{k}=\frac{\Phi^{\prime }(\nabla_{x}D_{t|k})}{2\nabla_{x}D_{t|k}}.$$

$P_{t-1}^{⊥}$ is the projector onto $E_{{\hat{D}}_{t-1}}^{⊥}$.

##### Spatio-temporal modelling

Apart from imposing temporal smoothness constraints, Galka *et. al*. [35] solved the inverse problem by recasting it as a spatio-temporal state space model which they solve by using Kalman filtering. The computational complexity of this approach that arises due to the high dimensionality of the state vector was addressed by decomposing the model into a set of coupled low-dimensional problems requiring a moderate computational effort. The initial state estimates for the Kalman filter are provided by LORETA. It is shown that by choosing appropriate dynamical models, better solutions than those obtained by the instantaneous inverse solutions (such as LORETA) are obtained.

##### 3.1.2 The Backus-Gilbert method

The Backus-Gilbert method [5,7,36] consists of finding an approximate inverse operator **T **of **G **that projects the EEG data **M **onto the solution space in such a way that the estimated primary current density ${\hat{D}}_{BG}$ = **TM**, is closest to the real primary current density inside the brain, in a least square sense. This is done by making the 1 × *p *vector $R_{uv\gamma }^{T}=T_{u\gamma }^{T}G_{v}$ (*u*, *v *= 1, 2, 3 and *γ *= 1, ..., *p*) as close as possible to $\delta_{uv}I_{\gamma }^{T}$ where *δ *is the Kronecker delta and **I**_*γ *_is the *γ *th column of the *p *× *p *identity matrix. **G**_*v *_is a *N *× *p *matrix derived from **G **in such a way that in each row, only the elements in **G **corresponding to the *v*th direction are kept. The Backus-Gilbert method seeks to minimize the spread of the resolution matrix **R**, that is to maximize the resolving power. The generalized inverse matrix **T **optimizes, in a weighted sense, the resolution matrix.

We reproduce the discrete version of the Backus-Gilbert problem as given in [5]:

$$\underset{T_{u\gamma }}{\min}\{{\left[I_{\gamma }-G_{u}^{T}T_{u\gamma }\right]}^{T}W_{\gamma }^{BG}[I_{\gamma }-G_{u}^{T}T_{u\gamma }]+\sum_{v=1}^{3}\left(1-\delta_{vu})T_{u\gamma }^{T}G_{v}G_{v}^{T}T_{u\gamma }\right\}$$

under the normalization constraint: $T_{u\gamma }^{T}G_{u}1_{p}=1$. 1_*p *_is a *p *× 1 matrix consisting of ones.

One choice for the *p *× *p *diagonal matrix $W_{\gamma }^{BG}$ is:

$$\begin{array}{cc} {\left[W_{\gamma }^{BG}\right]}_{\alpha \alpha }=||v_{\alpha }-v_{\gamma }|{|}^{2}, & \forall \alpha ,\gamma =1,...,p \end{array}$$

where **v**_*i *_is the position vector of grid point *i *in the head model. Note that the first part of the functional to be minimized attempts to ensure correct position of the localized dipoles while the second part ensures their correct orientation.

The solution for this EEG Backus-Gilbert inverse operator is:

$$T_{u\gamma }=\frac{E_{u\gamma }^{†}L_{u}}{L_{u}^{T}E_{u\gamma }^{†}L_{u}}$$

where:

$$\begin{array}{c} L_{u}=G_{u}1_{p},E_{u\gamma }=C_{u\gamma }+\sum_{v=1}^{3}(1-\delta_{uv})F_{v},\\ C_{u\gamma }=G_{u}W_{\gamma }^{BG}G_{u}^{T},F_{v}=G_{v}G_{v}^{T}. \end{array}$$

'^†^' denotes the Moore-Penrose pseudoinverse.

#### 3.1.3 The weighted resolution optimization

An extension of the Backus-Gilbert method is called the Weighted Resolution Optimization (WROP) [37]. The modification by Grave de Peralta Menendez is cited in [5]. $W_{\gamma }^{BG}$ is replaced by $W_{1\gamma }^{GdeP}$ where

$${\left[W_{1\gamma }^{GdeP}\right]}_{ll}=||v_{l}-v_{\gamma }|{|}^{2}+\beta^{GdeP}.$$

The second part of the functional to be minimzed is replaced by

$$\sum_{v=1}^{3}\left(1-\delta_{uv}\right)T_{u\gamma }^{T}G_{v}W_{2\gamma }^{GdeP}G_{v}^{T}T_{u\gamma }$$

where

$${\left[W_{2\gamma }^{GdeP}\right]}_{ll}=||v_{l}-v_{\gamma }|{|}^{2}+\beta^{GdeP}+\alpha^{GdeP},$$

*α*^*GdeP *^and *β*^*GdeP *^are scalars greater than zero. In practice this means that there is more trade off between correct localization and correct orientation than in the above Backus-Gilbert inverse problem.

In this case the inverse operator is:

$$T_{u\gamma }=\beta^{GdeP}\{G_{u}W_{1\gamma }^{GdeP}G_{u}^{T}+\sum_{v=1}^{3}(1-\delta_{uv})G_{v}W_{2\gamma }^{GdeP}G_{v}^{T}{\}}^{†}G_{u}I_{\gamma }.$$

In [5] five different inverse methods (the class of instantaneous, 3D, discrete linear solutions for the EEG inverse problem) were analyzed and compared for noise-free measurements: minimum norm, weighted minimum norm, Backus-Gilbert, weighted resolution optimization (WROP) and LORETA. Of the five inverse solutions tested, only LORETA demonstrated the ability of correct localization in 3D space.

The WROP method is a family of linear distributed solutions including all weighted minimum norm solutions. As particular cases of the WROP family there are LAURA [26,38], a local autoregressive average which includes physical constraints into the solutions and EPI-FOCUS [38] which is a linear inverse (quasi) solution, especially suitable for single, but not necessarily point-like generators in realistic head models. EPIFOCUS has demonstrated a remarkable robustness against noise.

##### LAURA

As stated in [39] in a norm minimization approach we make several assumptions in order to choose the optimal mathematical solution (since the inverse problem is underdetermined). Therefore the validity of the assumptions determine the success of the inverse solution. Unfortunately, in most approaches, criteria are purely mathematical and do not incorporate biophysical and psychological constraints. LAURA (Local AUtoRegressive Average) [40] attempts to incorporate biophysical laws into the minimum norm solution.

According to Maxwell's laws of electromagnetic field, the strength of each source falls off with the reciprocal of the cubic distance for vector fields and with the reciprocal of the squared distance for potential fields. LAURA method assumes that the electromagnetic activity will occur according to these two laws.

In LAURA the current estimate is given by the following equation:

$${\hat{D}}_{LAURA}=W_{j}G^{T}{\left(GW_{j}^{-1}G^{T}+\alpha I_{N}\right)}^{-1}M$$

The **W**_*j *_matrix is constructed as follows:

1. Denote by $V_{i}$ the vicinity of each solution point defined as the hexahedron centred at the point and comprising at most $V_{max}$ = 26 points.

2. For each solution point denote by *N*_*k *_the number of neighbours of that point and by *d*_*ki *_the Euclidean distance from point *k *to point *i *(and vice versa).

3. Compute the matrix **A **using *e*_*i *_= 2 for scalar fields and *e*_*i *_= 3 for vector fields

$$A_{ii}=\frac{V_{max}}{N_{i}}\sum_{k\in V_{i}}d_{ki}^{-e_{i}}$$

and

$$A_{ik}=-d_{ki}^{-e_{i}}$$

4. The weight matrix **W**_*j *_is defined by:

**W**_*j *_= **P**^*T*^**P**

where:

**P **= **W**_*m*_**A **⊗ **I**_3_

where **I**_3 _is the 3 × 3 identity matrix and ⊗ denotes the Kronecker product. **W**_*m *_is a diagonal matrix formed by the mean of the norm of the three columns of the lead field matrix associated with the *i*th point.

#### 3.1.4 Shrinking methods and multiresolution methods

By applying suitable iterations to the solution of a distributed source model, a concentrated source solution may be obtained. Ways of performing this are explained in the next section.

##### S-MAP with iterative focusing

This modified version [27] of Spatial Regularization is dedicated to the recovery of focal sources when the spatial sampling of the cortical surface is sparse. The source space dimension is reduced by iterative focusing on the regions that have been previously estimated with significant dipole activity. An energy criterion is used which takes into consideration both the source intensities and its contribution to data:

*E *= 2*E*_*c *_+ *E*_*a*_

where *E*_*c *_measures the contribution of every dipole source to the data and *E*_*a *_is an indicator of dipole relative magnitudes. Sources with energy greater than a certain threshold are selected for the next iteration. The estimator at the *i*th iteration is given by

$${\hat{D}}_{i}=\Theta (G_{i-1},L({\hat{D}}_{i-1})).M$$

where **G**_*i *_is the column-reduced version of **G **and **Θ **is a *p*_*i *_≤ *p *by *N *matrix depending on the **G**_*i *_and priors computed from the previous source estimate ${\hat{D}}_{i-1}$. A similar approach was used in [31] where the source region was contracted several times but at each iteration, LORETA was used to estimate the source tomography.

##### Shrinking LORETA-FOCUSS

This algorithm combines the ideas of LORETA and FOCUSS and makes iterative adjustments to the solution space in order to reduce computation time and increase source resolution [?, 20]. Starting from the smooth LORETA solution, it enhances the strength of some prominent dipoles in the solution and diminishes the strength of other dipoles. The steps [20] are as follows:

1. The current density is computed using LORETA to get ${\hat{D}}_{LOR}$.

2. The weighting matrix **W **is constructed using (10), its initial value being given by $W_{0}=\text{diag}({\hat{D}}_{LOR})=\text{diag}({\hat{D}}_{0}(1),{\hat{D}}_{0}(2),...,{\hat{D}}_{0}(3p))$.

3. The current density ${\hat{D}}_{i}$ is computed using (9).

4. (Smoothing operation) The prominent nodes (e.g. those with values larger than 1% of the maximum value) and their neighbours are retained. The current density values on these prominent nodes and their neighbours are readjusted by smoothing, the new values being given by

$$\begin{array}{ccc} \frac{1}{s_{l}+1}\left(\hat{D}(l)+\sum_{u}\hat{D}(u)\right) & \forall u\text{ under constraint} & ||r_{l}-r_{u}||=d \end{array}$$

where *r*_*l *_is the position vector of the *l*th node and *s*_*l *_is the number of neighbouring nodes around the *l*th node with distance equal to the minimum inter-node distance *d*.

5. (Shrinking operation) The corresponding elements in $\hat{D}$ and **G **are retained and the matrix **M **= **D**$\hat{D}$ is computed.

6. Steps (2) to (5) are repeated until convergence.

7. The solution of the last iteration before smoothing is the final solution.

Steps (4) and (5) are stopped if the new solution space has fewer nodes than the number of electrodes or the solution of the current iteration is less sparse than that estimated by the previous iteration. Once steps (4) and (5) are stopped, the algorithm becomes a FOCUSS process. Results [20] using simulated noiseless data show that Shrinking LORETA-FOCUSS is able to reconstruct a three-dimensional source distribution with smaller localization and energy errors compared to Weighted Minimum Norm, the *L*_1 _approach and LORETA with FOCUSS. It is also 10 times faster than LORETA with FOCUSS and several hundred times faster than the *L*_1 _approach.

##### Standardized shrinking LORETA-FOCUSS (SSLOFO)

SSLOFO [41] combines the features of high resolution (FOCUSS) and low resolution (WMN, sLORETA) methods. In this way, it can extract regions of dominant activity as well as localize multiple sources within those regions. The procedure is similar to that in Shrinking LORETA-FOCUSS with the exception of the first three steps which are:

1. The current density is computed using sLORETA to get ${\hat{D}}_{sLOR}$.

2. The weighting matrix **W **is constructed using (10), its initial value being given by $W_{0}=\text{diag}({\hat{D}}_{sLOR})=\text{diag}({\hat{D}}_{0}(1),{\hat{D}}_{0}(2),...,{\hat{D}}_{0}(3p))$.

3. The current density ${\hat{D}}_{i}$ is computed using (9). The power of the source estimation is then normalized as

(12) $${\hat{D}}_{i}^{T}(l){\left\{{\left[R_{i}\right]}_{ll}\right\}}^{-1}D_{i}(l)$$

where $R_{i}=W_{i}W_{i}^{T}G^{T}{\left(GW_{i}W_{i}^{T}+\alpha I\right)}^{†}G$ and [**R**_*i*_]_*ll *_is the *l*th diagonal block of matrix **R**_*i*_.

In [41], SSLOFO reconstructed different source configurations better than WMN and sLORETA. It also gave better results than FOCUSS when there were many extended sources. A spatio-temporal version of SSLOFO is also given in [41]. An important feature of this algorithm is that the temporal waveforms of single/multiple sources in the simulation studies are clearly reconstructed, thus enabling estimation of neural dynamics directly from the cortical sources. Neither Shrinking LORETA-FOCUSS nor FOCUSS are able to accurately reconstruct the time series of source activities.

##### Adaptive standardized LORETA/FOCUSS (ALF)

The algorithms described above require a full computation of the matrix **G**. On the other hand, ALF [42] requires only 6%–11% of this matrix. ALF localizes sources from a sparse sampling of the source space. It minimizes forward computations through an adaptive procedure that increases source resolution as the spatial extent is reduced. The algorithm has the following steps:

1. A set of successive decimation ratios on the set of possible sources is defined. These ratios determine successively higher resolutions, the first ratio being selected so as to produce a targeted number of sources chosen by the user and the last one produces the full resolution of the model.

2. Starting with the first decimation ratio, only the corresponding dipole locations and columns in **G **are retained.

3. sLORETA (Equation(11)) is used to achieve a smooth solution. The source with maximum normalized power is selected as the centre point for spatial refinement in the next iteration, in which the next decimation ratio is applied. Successive iterations include sources within a spherical region at successively higher resolutions.

4. Steps 2 and 3 are repeated until the last decimation ratio is reached. The solution produced by the final iteration of sLORETA is used as initialization of the FOCUSS algorithm. Standardization (Equation(12)) is incorporated into each FOCUSS iteration as well.

5. Iterations are continued until there is no change in solution.

It is shown in [42] that the localization accuracy achieved is not significantly different than that obtained when an exhaustive search in a fully-sampled source space is made. A multiresolution framework approach was also used in [15]. At each iteration of the algorithm, the source space on the cortical surface was scanned at higher spatial resolution such that at every resolution but the highest, the number of source candidates was kept constant.

#### 3.1.5 Summary

Refering to Equation (8), Table 1 summarizes the different weight matrices used in the algorithms. Refering to Subsection 3.1.4, Table 2 summarizes the steps involved in the different iterative methods which were discussed.

**Table 1:** Summary of weighting strategies for the various non-parametric methods. For definition of notation, refer to the respective subsection.

Algorithm	Weight Matrix **W**
MNE	**I**_3_*p*

WMNE	**Ω **⊗ **I**_3_

LORETA	(**Ω **⊗ **I**_3_)Δ^*T*^Δ(**Ω **⊗ **I**_3_)

Quadratic Regularization	∇

LAURA	**W**_*m*_**A **⊗ **I**_3_

**Table 2:** Steps involved in the iterative methods

Iterative Method	Description
S-MAP with Iterative Focusing	Uses the S-MAP algorithm; an energy criterion is used to reduce the dimension of **G**; priors computed from the previous source estimate are used at each new iteration.

Shrinking LORETA-FOCUSS	LORETA solution computed; Weighting matrix **W **constructed; FOCUSS algorithm used to estimate $\hat{D}$; smoothing of current density values of prominent dipoles and their neighbours; shrinking of $\hat{D}$ and **G**; computation of **M **= **G**$\hat{D}$; process (computation of **W **etc.) repeated.

SSLOFOM	sLORETA solution computed; Weighting matrix **W **constructed; FOCUSS algorithm used to estimate $\hat{D}$; source estimation power is normalized; smoothing of current density values of prominent dipoles and their neighbours; shrinking of $\hat{D}$ and **G**; computation of **M **= **G**$\hat{D}$; process (computation of **W **etc.) repeated.

ALF	Decimation ratios are defined; first ratio is used to retain the corresponding dipole locations and columns of **G**; sLORETA computed; source with maximum normalized power selected as centre point for spatial refinement; next decimation ratio used; process repeated until last ratio is reached; final sLORETA solution used to initialize FOCUSS algorithm with standardization.

### 3.2 Parametric methods

Parametric Methods are also referred to as Equivalent Current Dipole Methods or Concentrated Source or Spatio-Temporal Dipole Fit Models. In this approach, a search is made for the best dipole position(s) and orientation(s). The models range in complexity from a single dipole in a spherical head model, to multiple dipoles (up to ten or more) in a realistic head model. Dynamic models take into consideration dipole changes in time as well. Constraints on the dipole orientations, whether fixed or variable, may be made as well.

#### 3.2.1 The non-linear least-squares problem

The best location and dipole moment (six parameters in all for each dipole) are usually obtained by finding the global minimum of the residual energy, that is the *L*_2_-norm ||*V*_*in *_- *V*_*model*_||, where *V*_*model *_∈ ℝ^*N *^represents the electrode potentials with the hypothetical dipoles, and *V*_*in *_∈ ℝ^*N *^represents the recorded EEG for a single time instant. This requires a non-linear minimization of the cost function ||**M **- **G**({**r**_*j*_, $r_{dip_{i}}$})**D**|| over all of the parameters ($r_{dip_{i}}$, **D**). Common search methods include the gradient, downhill or standard simplex search methods (such as Nelder-Mead) [43-46], normally including multi-starts, as well as genetic algorithms and very time-consuming simulated annealing [45,47,48]. In these iterative processes, the dipolar source is moved about in the head model while its orientation and magnitude are also changed to obtain the best fit between the recorded EEG and those produced by the source in the model. Each iterative step requires several forward solution calculations using test dipole parameters to compare the fit produced by the test dipole with that of the previous step.

#### 3.2.2 Beamforming approaches

Beamformers are also called spatial filters or virtual sensors. They have the advantage that the number of dipoles must not be assumed *a priori*. The output **y**(*t*) of the beamformer is computed as the product of a 3 × *N *(each Cartesian axis is considered) spatial filtering matrix **W**^*T *^with **m**(*t*), the *N *× 1 vector representing the signal at the array at a given time instant *t *associated with a single dipole source, i.e. **y**(*t*) = **W**^*T*^**m**(*t*). This output represents the neuronal activity of each dipole d in the best possible way at a given time *t*.

In beamforming approaches [6], the signals from the electrodes are filtered in such a way that only those coming from sources of interest are maintained. If the location of interest is **r**_*dip*_, the spatial filter should satisfy the following constraints:

$$W^{T}(r_{dip})G(r)=\{\begin{array}{cc} I, & ||r-r_{dip}||\leq \delta \\ 0, & ||r-r_{dip}||>\delta \end{array}$$

where **G**(**r**) = [**g**(**r**, **e**_*x*_), **g**(**r**, **e**_*y*_), **g**(**r**, **e**_*z*_)] is the *N *× 3 forward matrix for three orthogonal dipoles at location **r **having orientation vectors **e**_*x*_, **e**_*y *_and **e**_*z *_respectively, **I **is the 3 × 3 identity matrix and *δ *represents a small distance.

In linearly constrained minimum variance (LCMV) beamforming [49], nulls are placed at positions corresponding to interfering sources, i.e. neural sources at locations other than **r**_*dip *_(so *δ *= 0). The LCMV problem can be written as:

$$\begin{array}{ccc} \underset{W^{T}}{\min}Tr(C_{y}) & \text{subject to} & W^{T}(r_{dip})G(r_{dip})=I \end{array}$$

where **C**_**y **_= *E*[**yy**^*T*^] = **W**^*T*^**C**_**m**_**W **and **C**_**m **_= *E*[**mm**^*T*^] is the signal covariance matrix estimated from the available data. This means that the beamformer minimizes the output energy **W**^*T*^**C**_**m**_**W **under the constraint that only the dipole at **r**_*dip *_is active at that time. Minimization of variance optimally allocates the stop band response of the filter to attenuate activity originating at other locations. By applying Lagrange multipliers and completing the square (proof in Appendix), one obtains:

$$W(r_{dip})={\left[G{\left(r_{dip}\right)}^{T}C_{m}^{-1}G(r_{dip})\right]}^{-1}G{\left(r_{dip}\right)}^{T}C_{m}^{-1}.$$

The filter **W**(**r**_*dip*_) is then applied to each of the vectors **m**(*t*) in **M **so that an estimate of the dipole moment at **r**_*dip *_is obtained. To perform localization, an estimation of the variance or strength $V\hat{a}r$(**r**_*dip*_) of the activity as a function of location is calculated. This is the value of the cost function *Tr*{**W**^*T*^(**r**_*dip*_)**C**_**m**_**W**(**r**_*dip*_)} at the minimum, equal to $Tr\{{\left[G^{T}(r_{dip})C_{m}^{-1}G(r_{dip})\right]}^{-1}\}$.

This approach can produce an estimate of the neural activity at any location by changing the location **r**_*dip*_. It assumes that any source can be explained as a weighted combination of dipoles. Hence the geometry of sources is not restricted to points but may be distributed in nature according to the variance values. Moreover, this approach does not require prior knowledge of the number of sources and anatomical information is easily included by evaluating $V\hat{a}r$(**r**_*dip*_) only at physically realistic source locations.

The resolution of detail obtained by this approach depends on the filter's passband and on the SNR (signal to noise ratio defined as the ratio of source variance to noise variance) associated with the feature of interest. To minimimize the effect of low SNRs, the estimated variance is normalized by the estimated noise spectral spectrum to obtain what is called the neural activity index:

$$V\hat{a}r_{N}(r_{dip})=\frac{V\hat{a}r(r_{dip})}{Tr\{{\left[G^{T}(r_{dip})Q^{-1}G(r_{dip})\right]}^{-1}\}}$$

where **Q **is the noise covariance matrix estimated from data that is known to be source free.

Sekihara *et. al *[50] proposed an 'eigenspace projection' beamformer technique in order to reconstruct source activities at each instant in time. It is assumed that, for a general beamformer, the matrix **W **= [**w**_*x*_, **w**_*y*_, **w**_*z*_] where the column weight vectors **w**_*x*_, **w**_*y *_and **w**_*z*_, respectively, detect the *x*, *y *and *z *components of the source moment to be determined and are of the form

$$w_{\mu }=\frac{C_{m}^{-1}G(r_{dip}){\left[G^{T}(r_{dip})C_{m}^{-1}G(r_{dip})\right]}^{-1}f_{\mu }}{\sqrt{f_{\mu }^{T}\Omega f_{\mu }}}$$

where *μ *= *x*, *y *or *z*, **f**_*x *_= [1, 0, 0]^*T*^, **f**_*y *_= [0,1 0]^*T*^, **f**_*z *_= [0, 0, 1]^*T *^and

$$\Omega ={\left[G^{T}(r_{dip})C_{m}^{-1}G(r_{dip})\right]}^{-1}G^{T}C_{m}^{-2}G(r_{dip}){\left[G^{T}(r_{dip})C_{m}^{-1}G(r_{dip})\right]}^{-1}$$

The weight vectors for the proposed beamformer, ${\tilde{w}}_{\mu }$, are derived by projecting the weight vectors **w**_*μ *_onto the signal subspace of the measurement covariance matrix:

$${\tilde{w}}_{\mu }=E_{S}E_{S}^{T}w_{\mu }$$

where **E**_*S *_is the matrix whose columns consist of the signal-level eigenvectors of **C**_**m**_. This beamformer, when tested on Magnetoencephalography (MEG) experiments, not only improved the SNR considerably but also the spatial resolution. In [50], it is further extended to a prewhitened eigenspace projection beamformer to reduce interference arising from background brain activities.

#### 3.2.3 Brain electric source analysis (BESA)

In a particular dipole-fit model called Brain Electric Source Analysis (BESA) [27], a set of consecutive time points is considered in which dipoles are assumed to have fixed position and fixed or varying orientation. The method involves the minimization of a cost function that is a weighted combination of four criteria: the Residual Variance (RV) which is the amount of signal that remains unexplained by the current source model; a Source Activation Criterion which increases when the sources tend to be active outside of their *a priori *time interval of activation; an Energy Criterion which avoids the interaction between two sources when a large amplitude of the waveform of one source is compensated by a large amplitude on the waveform of the second source; a Separation Criterion that encourages solutions in which as few sources as possible are simultaneously active.

#### 3.2.4 Subspace techniques

We now consider parametric methods which process the EEG data prior to performing the dipole localization. Like beamforming techniques, the number of dipoles need not be known *a priori*. These methods can be more robust since they can take into consideration the signal noise when performing dipole localization.

##### Multiple-signal Classification algorithm (MUSIC)

The multiple-signal Classification algorithm (MUSIC) [6,51] is a version of the spatio-temporal approach. The dipole model can consist of fixed orientation dipoles, rotating dipoles or a mixture of both. For the case of a model with fixed orientation dipoles, a signal subspace is first estimated from the data by finding the singular value decomposition (SVD) [8]**M **= **UΣV**^*T *^and letting **U**_*S *_be the signal subspace spanned by the *p *first left singular vectors of **U**. Two other methods of estimating the signal subspace, claimed to be better because they are less affected by spatial covariance in the noise, are given in [52]. The first method involves prewhitening of the data matrix making use of an estimate of the spatial noise covariance matrix. This means that the data matrix **M **is transformed so that the spatial covariance matrix of the transformed noise matrix is the identity matrix. The second method is based on an eigen decomposition of a matrix product of stochastically independent sweeps. The MUSIC algorithm then scans a single dipole model through the head volume and computes projections onto this subspace. The MUSIC cost function to be minimized is

$$\frac{||P_{S}^{⊥}g(r,e)|{|}^{2}}{||g(r,e)|{|}^{2}}$$

where $P_{S}^{⊥}=I-(U_{S}U_{S}^{T})$ is the orthogonal projector onto the noise subspace, **r **and **e **are position and orientation vectors, respectively. This cost function is zero when **g**(**r**, **e**) corresponds to one of the true source locations and orientations, **r **= $r_{dip_{i}}$ and **e **= $e_{d_{i}}$, *i *= 1, ..., *p*. An advantage over least-squares estimation is that each source is found in turn, rather than searching simultaneously for all sources.

In MUSIC, errors in the estimate of the signal subspace can make localization of multiple sources difficult (subjective) as regards distinguishing between 'true' and 'false' peaks. Moreover, finding several local maxima in the MUSIC metric becomes difficult as the dimension of the source space increases. Problems also arise when the subspace correlation is computed at only a finite set of grid points.

Recursive MUSIC (R-MUSIC) [53] automates the MUSIC search, extracting the location of the sources through a recursive use of subspace projection. It uses a modified source representation, referred to as the spatio-temporal independent topographies (IT) model, where a source is defined as one or more nonrotating dipoles with a single time course rather than an individual current dipole. It recursively builds up the IT model and compares this full model to the signal subspace.

In the recursively applied and projected MUSIC (RAP-MUSIC) extension [54,55], each source is found as a global maximizer of a different cost function. Assuming **g**(**r**, **e**) = **h**(**r**)**e**, the first source is found as the source location that maximizes the metric

$${\hat{r}}_{1}=\arg\underset{r}{\max}(subcorr{\left(h(r),U_{S}\right)}_{1})$$

over the allowed source space, where **r **is the nonlinear location parameter. The function *subcorr*(**h**(**r**), **U**_*S*_)_1 _is the cosine of the first principal angle between the subspaces spanned by the columns of **h**(**r**) and **U**_*S *_given by:

$$subcorr{\left(h(r),U_{S}\right)}_{1}^{2}=\frac{\left(h{\left(r\right)}^{T}U_{S},U_{S}^{T}h(r)\right)}{\left(h{\left(r\right)}^{T}h(r)\right)}$$

The *k*-th recursion of RAP-MUSIC is

(13) $${\hat{r}}_{k}=\arg\underset{r}{\max}(subcorr{\left(\Pi_{{\hat{G}}_{k-1}}^{⊥}h(r),\Pi_{{\hat{G}}_{k-1}}^{⊥}U_{S}\right)}_{1})$$

where ${\hat{G}}_{k-1}\equiv [g({\hat{r}}_{1},{\hat{e}}_{1})...g({\hat{r}}_{k-1},{\hat{e}}_{k-1})]$ is formed from the array manifold estimates $\left(g({\hat{r}}_{i},{\hat{e}}_{i})=h({\hat{r}}_{i}){\hat{e}}_{i}\right)$ of the previous *k *- 1 recursions and $\Pi_{{\hat{G}}_{k-1}}^{⊥}\equiv (I-{\hat{G}}_{k-1}{\left({\hat{G}}_{k-1}^{T}{\hat{G}}_{k-1}\right)}^{-1}{\hat{G}}_{k-1}^{T})$ is the projector onto the left-null space of ${\hat{G}}_{k-1}$. The recursions are stopped once the maximum of the subspace correlation in (13) drops below a minimum threshold.

A key feature of the RAP-MUSIC algorithm is the orthogonal projection operator which removes the subspace associated with previously located source activity. It uses each successively located source to form an intermediate array gain matrix and projects both the array manifold and the estimated signal subspace into its orthogonal complement, away from the subspace spanned by the sources that have already been found. The MUSIC projection to find the next source is then performed in this reduced subspace. Other sequential subspace methods besides R-MUSIC and RAP-MUSIC are S-MUSIC and IES-MUSIC [54]. Although they all find the first source in the same way, in these latter methods the projection operator is applied just to the array manifold, rather than to both arguments as in the case of RAP-MUSIC.

##### FINES subspace algorithm

An alternative signal subspace algorithm [56] is FINES (First Principal Vectors). This approach, used in order to estimate the source locations, employs projections onto a subspace spanned by a small set of particular vectors (FINES vector set) in the estimated noise-only subspace instead of the entire estimated noise-only subspace as in the case of classic MUSIC.

In FINES the principal angle between two subspaces is defined according to the closeness criterion [56]. FINES creates a vector set for a region of the brain in order to form a projection operator and search for dipoles in this specific region.

An algorithmic description of the FINES algorithm can be found in [56]. Simulation results in [56] show that FINES produces more distinguishable localization results than classic MUSIC and RAP-MUSIC even when two sources are very close spatially.

#### 3.2.5 Simulated annealing and finite elements

In [47], an objective function based on the current-density boundary integral associated with standard finite-element formulations in two dimensions is used instead of measured potential differences, as the basis for optimization performed using the method of simulated annealing. The algorithm also enables user-defined target search regions to be incorporated. In this approach, the optimization objective is to vary the modelled dipole such that the Neumann boundary condition is satisfied, that is, the current density at each electrode approaches zero.

$$\min\sum_{t=1}^{N}C{\left(x_{p},y_{p},\theta_{p},d_{p}\right)}_{l}=\min\sum_{l=1}^{N}{\left(\oint \hat{n}.J_{l}\psi_{l}ds\right)}^{2}$$

where *C*(*x*_*p*_, *y*_*p*_, *θ*_*p*_, *d*_*p*_)_*l *_is the objective function associated with the *l*th electrode resulting from *p *dipoles, *N *is the number of electrodes, *J*_*l *_is the current density associated with the *l*th electrode, *ψ*_*l *_represents the weighting function associated with the *l*th electrode and $\hat{n}$ is the outward-pointing normal direction to the boundary of the problem domain. This formulation allows for the single calculation of the inverse or preconditioner matrix in the case of direct or iterative matrix solvers, respectively, which is a significant reduction in the computational time associated with 3-D finite element solutions.

#### 3.2.6 Computational intelligence algorithms

##### Neural networks

Since the inverse source localization problem can be considered a minimization problem – find the optimal coordinates and orientation for each dipole – the optimization can be performed with an artificial neural network (ANN) based system.

The main advantage of neural network approaches [57] is that once trained, no further iterative process is required. In addition, although iterative methods are shown to be better in noise free environments, ANN performs best in environments with low signal to noise ratio [58]. Therefore ANNs seem to be more noise robust. In any case, many research works [59-67] claim a localization error in ANN methods of less than 5%.

**Figure 3:**
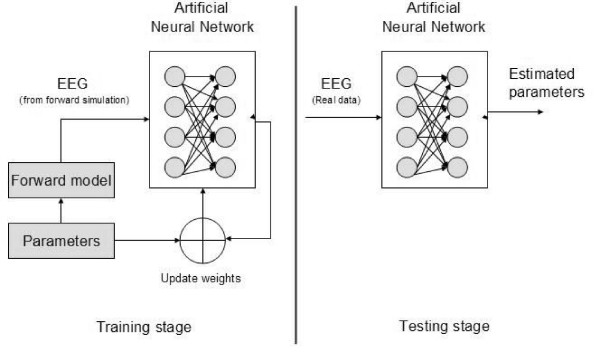
General block diagram for an artificial neural network system used for inverse source localization.

A general ANN system for EEG source localization is illustrated in Figure 3. According to [65], the number of neurons in the input layer is equal to the number of electrodes and the features at the input can be directly the values of the measured voltage. The network also consists of one or two hidden layers of *N *neurons each and an output layer made up of six neurons, 3 for the coordinates and 3 for dipole components. In addition each hidden layer neuron is connected to the output layer with weights equal to one in order to permit a non-zero threshold of the activation function. Weights of inter connections are determined after the training phase where the neural network is trained with preconstructed examples from forward modeling simulations.

##### Genetic algorithms

An alternative way to solve the inverse source localization problem as a minimization problem is to use genetic algorithms. In this case dipoles are modelled as a set of parameters that determine the orientation and the location of the dipole and the error between the projected potential and the measured potentials is minimized by genetic algorithm evolutionary techniques.

The minimization operation can be performed in order to localize multiple sources either in the brain [68] or in Independent Component backprojections [69,70]. If component back-projections are used, the correlation between the projected model and the measured one will have to be minimized rather than the energy of the difference.

**Figure 4:**
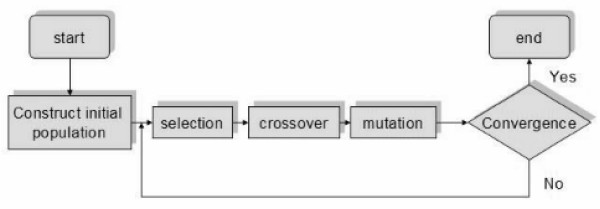
Genetic algorithm schema.

Figure 4 shows how the minimization approach develops. An initial population is created, this being a set of potential solutions. Every solution of the set is encoded e.g. binary code and then a new population is created with the application of three operators: selection, crossover and mutation. The procedure is repeated until convergence is reached.

#### 3.2.7 Estimation of initial dipoles for parametric methods

In most parametric methods, the final result is extremely dependent on the initial guess regarding the number of dipoles to estimate and their initial locations and orientations. Estimates can be obtained as explained below.

##### The optimal dipole

In this model, any point inside the sphere has an associated optimal dipole [71], which fits the observed data better than any other dipole that has the same location but different orientation. The unknown parameters of an optimal dipole are the magnitude components *d*_*x*_, *d*_*y *_and *d*_*z *_(which is a linear least-squares problem).

There are two possible ways for finding the required optimal dipoles. The first is a minimization iteration, where a few optimal dipoles are found in an arbitrary region. The steepest slope with respect to the spatial coordinate is then found and a new search region is obtained. Optimal dipoles in that region are found, a new slope is determined and so on, until the best of all optimal dipoles is found.

The second way is to find optimal dipoles at a dense grid of points that covers the volume in which the dipolar source is expected to be. The best of all the optimal dipoles is the chosen optimal dipole.

##### Stagnant dipoles

Stagnant dipoles [10] have only time varying amplitude (dipole magnitude). So the number of unknowns is *p*(5 + *T*) where *p *is the number of dipoles and *T *is the number of time-samples. In this model, the minimum number of dipoles *L *(assumed to be smaller than *N *or *T*) required to describe the observed responses adequately was determined. The resulting residual between the data matrix **M **and the corresponding model predictions $\hat{M}$ is expressed as

$$Tr[(M-\hat{M}){\left(M-\hat{M}\right)}^{T}]$$

where $\hat{M}$ is a *N *× *T *matrix of rank *L*. It is found that the resulting residual will never exceed the true residual *H*:

$$H\geq \underset{rank(\hat{M})=L}{\min}Tr[(M-\hat{M}){\left(M-\hat{M}\right)}^{T}].$$

This is equivalent to

$$H\geq \sum_{k=L+1}^{min(N,T)}\lambda_{k}^{2}$$

where *λ*_*k *_are the singular values of **M**, arranged in decreasing order. Hence more sources haveto be included in the model till the lower bound is smaller than the noise level *H*.

## 4 Performance analysis

In this section we do a comparative review of the performance of different methods as recorded in the literature and subsequently report our own Monte-Carlo comparative experiments. Various measures have been used to determine the quality of localization obtained by a given algorithm and each measure represents some performance aspect of the algorithm such as localization error, smoother error, generalization etc. In the following we review a number of measures that capture various characteristics of localization performance.

For a single point source, localization error is the distance between the maximum of the estimated solution and the actual source location [20,27,32]. An extension of this which takes into consideration the magnitudes of other sources besides the maximum is the formula

$$\sum_{n=1}^{p}|\hat{D}(n)|d_{n}$$

where *d*_*n *_= |$r_{dip_{n}}$ - **r**_*dip*_| is the distance from the *n*th element to the true source [30].

The energy error [20], which describes the accuracy of the dipole magnitudes, is defined as

$$E_{energy}=\left|1-\frac{\left||{\hat{D}}_{max}|\right|}{\left||D|\right|}\right|$$

where $\left||{\hat{D}}_{max}|\right|$ is the power of the maxima in the estimated current distribution and ||**D**|| is the power of the simulated point source.

Baillet and Garnero [4] also determine the Data Fit and Reconstruction Error to evaluate the performance of their algorithms where Data Fit is defined as

$$\frac{\left||M-G\hat{D}|\right|}{\left||M|\right|}$$

and the Reconstruction Error (in which orientation of the dipoles is taken into consideration) is defined as

$$\frac{\left||D-\hat{D}|\right|}{\left||D|\right|}.$$

Baillet [27] also estimates the distance between the actual source location and the position of the centre of gravity of the source estimate by:

$$\left|\frac{\sum_{n=1}^{p}\left|\hat{D}(n)||r_{dip_{n}}\right|}{\sum_{n=1}^{p}\left|\hat{D}(n)\right|}-|r_{dip}|\right|$$

where $r_{dip_{n}}$ is the location of the *n*th source with intensity $\hat{D}$(*n*) and **r**_*dip *_is the original source location.

*E*_*spurious *_[27] is defined as the relative energy contained in spurious or phantom sources with regards to the original source energy.

For two dipoles [30], the true sources are first subtracted from the reconstructions. The error is:

$$\sum_{n=1}^{p}\left|\hat{D}(n)|(s_{n1}d_{n1}+s_{n2}d_{n2}\right)$$

where *d*_*n*1 _and *d*_*n*2 _are the distances from each element to the corresponding source, *s*_*n*1 _and *s*_*n*2 _were used to reduce the penalty due to the distance between a source and a part of the solution near the other source. This means that a large dipole magnitude solution near to one source should not give rise to an improper error estimate due to the potentially large distance to the other source. *s*_*n*1 _can be chosen in the following way:

$$s_{n1}=\{\begin{array}{cc} \begin{array}{rr} \beta d_{n2} & (\text{where }\beta >0),\\ & 1, \end{array} & \begin{array}{l} \begin{array}{cc} \text{for} & d_{n2}<\text{some number} \end{array}\\ \text{otherwise} \end{array} \end{array}$$

and similarly for *s*_*n*2 _using *d*_*n*1_.

In [72], Yao and Dewald used three indicators to determine the accuracy of the inverse solution:

1. the error distance (ED) which is the distance between the true and estimated sources defined as

(14) $$\frac{1}{N_{I}}\sum_{i}^{N_{I}}\underset{j}{\min}\{||{\hat{r}}_{dip_{i}}-r_{dip_{j}}||\}+\frac{1}{N_{J}}\sum_{j\in J}^{N_{J}}\underset{i}{\min}\{||r_{dip_{j}}-{\hat{r}}_{dip_{i}}||\}.$$

$r_{dip_{i}}$ is the real dipole location of the simulated EEG data. ${\hat{r}}_{dip_{i}}$ is the source location detected by the inverse calculation method. *i *and *j *are the indices of locations of estimated and the actual sources. *N*_*I *_and *N*_*J *_are the total numbers of estimated and undetected sources respectively. In current distributed-source reconstruction methods, a source location was defined as the location where the current strength was greater than a set threshold value. The first term in (14) calculates the mean of the distance from each estimated source to its closest real source. The corresponding real source is then marked as a detected source. All the undetected real sources made up the elements of data set *J*. The second term calculates the mean of the distance from each of the undetected sources to the closest estimated sources.

2. the percentage of undetected source number (PUS) defined as $\frac{N_{un}}{N_{real}}$ where *N*_*un *_and *N*_*real *_are the numbers of undetected and real sources respectively. An undetected source is defined as a real source whose location to its closest estimated source is larger than 0.6 times the unit distance of the source layer.

3. the percentage of falsely-detected source number (PFS) defined as $\frac{N_{false}}{N_{estimated}}$ where *N*_*false *_and *N*_*estimated *_are the numbers of falsely-detected and estimated sources respectively. A falsely-detected source is defined as an estimated source whose location to its closest real source is larger than 0.6 times the unit distance of the source layer.

The accuracy with which a source can be localized is affected by a number of factors including source-modeling errors, head-modeling errors, measurement-location errors and EEG noise [73]. Source localization errors depend also on the type of algorithm used in the inverse problem. In the case of *L*_1 _and *L*_2 _minimum norm approaches, the localization depends on several factors: the number of electrodes, grid density, head model, the number and depth of the sources and the noise levels.

In [3], the effects of dipole depth and orientation on source localization with varying sets of simulated random noise were investigated in four realistic head models. It was found that if the signal-to-noise ratio is above a certain threshold, localization errors in realistic head models are, on average, the same for deep and superficial sources and no significant difference in accuracy for radial or tangential dipoles is to be expected. As the noise increases, localization errors increase, particularly for deep sources of radial orientation. Similarly, in [46] it was found that the importance of the realistic head model over the spherical head model reduces by increasing the noise level. It has also been found that solutions for multiple-assumed sources have greater sensitivity to small changes in the recorded EEGs as compared to solutions for a single dipole [73].

### 4.1 Literature review of performance results of different inverse solutions

This section provides a review of the performance results of the different inverse solutions that have been described above and cites literature works where some of these solutions have been compared.

In [5], Pascual-Marqui compared five state-of-the-art parametric algorithms which are the minimum norm (MN), weighted minimum norm (WMN), Low resolution electromagnetic tomography (LORETA), Backus-Gilbert and Weighted Resolution Optimization (WROP). Using a three-layer spherical head model with 818 grid points (intervoxel distance of 0.133) and 148 electrodes, the results showed that on average only LORETA has an acceptable localization error of 1 grid unit when simulating a scenario with a single source. The other four inverse solutions failed to localize non-boundary sources and hence the author states that these solutions cannot be classified as tomographies as they lack depth information. When comparing MN solutions and LORETA solutions with different *L*_*p *_norms, Jun Yao *et al*. [72] have also found out that LORETA with the *L*_1 _norm gives the best overall estimation. In this analysis a Boundary Element Method (BEM) realistic head model was used and the simulated data consisted of three different datasets, designed to evaluate both localization ability and spatial resolution. Unlike the data in [5], this simulated data included noise and the SNR was set to 9–10 dB in each case. The results provided were the averaged results over 10 time samples. In [72] these current distribution methods were also compared to dipole methods where only a number of discrete generators are active. Results showed that although these methods give relatively low error distance measures, meaning that a priori knowledge of the number of dipoles could improve the inverse results, a medial and posterior shift occurred for all the three datasets. This shift was not present for current distribution methods. Jun Yao *et al*. have also used the percentage of undetected sources (PUS) as a measure to compare solutions. LORETA with the *L*_1 _norm resulted in a significantly smaller and less variable PUS when compared to all other methods tested in this paper [72]. The techniques were also applied to real data and once again the same approach gave qualitatively superior results which match those obtained with other neuroimaging techniques or cortical recordings.

Pascual-Marqui has also tested the effect on localization when the simulated data is made up of two sources [5]. In this case the LORETA and WROP solutions were used to localize these two sources, one of which was placed very deep. LORETA identified both sources but gave a blurred result, hence its name low resolution, but WROP did not succeed in identifying the two sources. MN, WMN and Backus-Gilbert gave a similar performance. From this analysis, Pascual-Marqui concluded that LORETA has the minimum necessary condition of being a tomography, however this does not imply that any source distribution can be identified suitably with LORETA [5].

In [32], Pascual-Marqui developed another algorithm called sLORETA. The name itself gives the impression that this is an updated version of LORETA but in fact it is based on the MN solution. The latter is known to suffer considerably when trying to localize deep sources. sLORETA handles this limitation by standardizing the MN estimates and basing localization inference on these standardized estimates [32]. When using a 3-layer spherical head model registered to the Talairach human brain atlas and assuming 6340 voxels with 5 *mm *resolution, sLORETA was found to give zero localization error for noiseless, single source simulations. In this case the solution space was restricted to the cortical gray matter and hippocampus areas. The same algorithm was tested on noisy data with noise scalp field standard deviation equal to 0.12 and 0.082 times the source with lowest scalp field standard deviation. The regularization parameter was in this case estimated using cross-validation. When compared to the Dale method, sLORETA was found to have the lowest localization errors and the lowest amount of blurring [32].

Another inverse solution considered as a complement to LORETA is VARETA [34]. This is a weighted minimum norm solution in which a regularization parameter that varies spatially is used. In LORETA this regularization parameter is a constant. In VARETA this parameter is altered to incorporate both concentrated and distributed sources. In [34], this inverse solution is compared to the FOCUSS algorithm [30]. The latter is a linear estimation method based on recursive, weighted norm minimization. When tested on noise-free simulations made up of a single or multi-source, FOCUSS achieved correct identification but the algorithm is initialization dependent. Iteratively it changes the weights based on the strength of the solution data in the last iteration and it converges to a set of concentrated sources which are equal in number to the number of electrodes. VARETA on the other hand is based on a Bayesian approach and does not necessarily converge to a set of dipoles if the simulated sources are distributed [34].

In [20], Hesheng Liu *et al*. developed another algorithm based on the foundation of LORETA and FOCUSS. This algorithm, called Shrinking LORETA FOCUSS (SLF) was tested on single and multi-source reconstruction and was compared to three other inverse solutions, mainly WMN, L1-NORM and LORETA-FOCUSS. In all scenarios considered, consisting of a number of sources organized in different arrangements and having different strengths and positions, SLF gave the closest solution to what was expected. WMN often gave a very blurred result, L1-NORM resulted in spurious sources or solutions with blurred images and incorrect amplitudes and LORETA-FOCUSS gave generally a high resolution but some sources were sometimes lost or had varying magnitude. LORETA-FOCUSS was found to have a localization error which was 3.2 times larger than that of SLF and an energy error which is 11.6 times larger. SLF is based on the assumption that the neuronal sources are both focal and sparse. If this is not the case and sources are distributed over a large area, then a low resolution solution as that offered by LORETA would be more appropriate [20].

Hesheng Liu [41] states that in situations where few sources are present and these are clustered, high resolution algorithms such as MUSIC can give better results. Solutions such as FOCUSS are also capable of reconstructing space sources. The performance of these algorithms degrades when the sources are large in number and extended in which case low resolution solutions such as LORETA and sLORETA are better. To achieve satisfactory results in both circumstances Hesheng Liu developed the SSLOFO algorithm [41]. Apart from localizing the sources with relatively high spatial resolution this algorithm gives also good estimates of the time series of the individual sources. Algorithms such as LORETA, sLORETA, FOCUSS and SLF fail to recover this temporal information satisfactorily. When compared with sLORETA for single source reconstruction, the mean error for SSLOFO was found to be 0 and the mean energy error was 2.99%. The mean error for sLORETA is also 0 but the mean energy error goes up to 99.55% as sLORETA has very poor resolution and a high point spread function [41]. In this same paper the authors have also analyzed the effect of noise. Noise levels ranging from 0 to 30 dB were considered and the mean localization error over a total of 2394 voxel positions was found. WMN and FOCUSS both resulted in large localization errors. sLORETA compared well with SSLOFO for a single source especially when the level of noise was high. When it comes to temporal resolution however, SSLOFO gave the best results. WMN mixed signals coming from nearby sources, sLORETA can only give power values and FOCUSS cannot produce a continuous waveform [41].

Another technique similar to SSLOFO in that it can capture spatial and temporal information is the ST-MAP [4]. If only spatial information is taken into account, the so called S-MAP succeeds in recovering most edges, preserving the global temporal shape but with possible sharp magnitude variations. The ST-MAP however gives a much smoother reconstruction of dipoles which helps stabilize the algorithm and reduce the computation time by 22% when compared to the S-MAP. These results were compared to those obtained by Quadratic Regularization (QR) and LORETA and the error measures were based on the data fit and reconstruction error. S-MAP and ST-MAP were both found to be superior to these algorithms as they give much smoother solutions and reasonably lower reconstruction errors [4].

Since the inverse problem in itself is underdetermined, most solutions use a mathematical criteria to find the optimal result. This however leaves out any reliable biophysical and psychological constraints. LAURA takes this into consideration and incorporates biophysical laws in the minimum norm solutions (MN, WMN, LORETA and EPIFOCUS [38]) and the simulation showed that EPIFOCUS and LAURA outperform LORETA. This comparison was done by measuring the percentage of dipole localization error less than 2 grid points.

Another solution to the inverse problem which was mentioned in Section 3.2.1 is BESA. This technique is very sensitive to the initial guess of the number of dipoles and therefore is highly dependent on the level of user expertise. In [74] it is shown that the grand average location error of 9 subjects who were familiar with evoked potential data was 1.4 cm with a standard deviation of 1 cm.

Rather than finding all possible sources simultaneously as is the case with direct least squares methods such as MN and LORETA, another class of inverse solutions exist based on signal subspace decomposition. A well known technique falling within this class is the multiple signal Classification (MUSIC) algorithm and its variants. These algorithms were developed because generally solutions were based on fitting a multiple modeling technique to a single time sample of EEG data [53]. Processing the whole length of data available then resulted in a large set of unknown parameters. Furthermore many techniques have the problem of getting trapped in local minima when minimizing their cost function. MUSIC and its variants were developed to overcome these problems and this is achieved by scanning a single dipole through a voxel grid placed within the 3D head model and working out the forward model at each voxel within the grid, projected against a signal subspace computed for that EEG data. Locations within this grid where the source model gave the best projections onto the signal subspace correspond to the dipole positions. In [54] a conventional two source uniform linear array example was used to compare various versions of MUSIC. A Monte Carlo test was carried out by allowing various runs to find each individual source. For uncorrelated sources all algorithms (MUSIC, S-MUSIC, IES-MUSIC, R-MUSIC and RAP-MUSIC) gave similar results but different performances were then observed as the level of correlation increased. At a correlation coeficient of 0.7, IES-MUSIC and RAP-MUSIC were found to have an RMS error around 25% better than that of MUSIC and S-MUSIC and 50% better than R-MUSIC. As the correlation increases RAP-MUSIC was found to give the best performance but at a value of 0.975 all methods experienced comparable difficulties in estimating the sources. RAP-MUSIC has the added advantage that it provides an automatic way of terminating the search for additional sources when the signal subspace is overestimated [54].

Another comparison with MUSIC and RAP MUSIC was done by using FINES, another signal subspace algorithm. In [56] a two-dipole simulated dataset in both noise-free and noisy environments was used for this comparison. In the noise-free case, FINES performs better than MUSIC with 0.2 mm and 1 mm lower error for the two dipoles respectively. When noise was added, FINES was still found to be superior than both MUSIC and RAP-MUSIC. The localization error for a SNR of 14 dB was found to be 1.4 mm and 1.5 mm lower when using FINES. In [75], He and Ding applied a similar analysis but this time using a realistic head model. The results are consistent with [56] showing that FINES is superior in the noisy scenario.

The literature review above shows that various comparisons have been performed between groups of inverse solutions. In the following subsection we would like to contribute to this comparative study by discussing the results obtained when performing a Monte-Carlo analysis to compare four non-parametric approaches of solving the EEG inverse problem. To our knowledge, such an analysis has not yet been carried out in the literature.

### 4.2 Monte Carlo performance analysis of non-parametric inverse solutions

Four widely used non-parametric approaches of solving the inverse problem were compared using a Monte-Carlo analysis where at each simulated dipole position (108 positions considered in total) a total of 100 source localization trials were performed. The effects of regularization and noise on each respective inverse solution were also analyzed.

#### 4.2.1 Inverse solutions

The four inverse solutions compared here were all implemented in MATLAB and included:

• Weighted Minimum Norm (WMN)

• Low Resolution Electromagnetic Tomography (LORETA)

• Standardized LORETA (sLORETA)

• Shrinking LORETA-FOCUSS (SLF)

These techniques were described in detail in earlier sections, thus they will not be described again here. For the SLF algorithm, however, the authors would like to point out that during the smoothing process, nodes at the boundary of the solution space were not smoothed as they have a limited amount of neighbours attributed to them. Also the recursive procedure was repeated until one of the following conditions was true: i) the number of prominent nodes in the solution space is less than the number of sensors, ii) the difference between the norms of consecutive current densities is less than 0.001, iii) the number of prominent nodes increases from one iteration to the next.

#### 4.2.2 Simulated data

A three-layer spherical head model with the properties as shown in Table 3 was considered:

**Table 3:** Properties of the 3-layer spherical head model

Radius of scalp	8 cm
Radius of skull	8/1.06 cm

Radius of cortex	8/1.15 cm

Conductivity of scalp	2860 mS/m

Conductivity of skull	2860/80 mS/m

Conductivity of cortex	2860 mS/m

Within the cortex, a grid made up of 755 voxels with inter-voxel distance of 1 cm was used. This choice was similar to that in [5] where the assumed unit spherical head model consisted of a grid made up of 818 voxels with an inter-voxel distance of 0.133. Higher resolutions of 7 mm and 5 mm were used in [20] and [32] respectively but the Monte Carlo analysis performed here placed some computational constraints restricting the choice of a finer resolution. Furthermore, in this analysis singular radial dipoles were considered. These dipoles had unit magnitude and were placed at each of 108 equally distributed positions out of the available 755 positions. For a radial dipole with Cartesian coordinates (*a*, *b*, *c*) and a sphere with center at (0, 0, 0), the dipole moment *e*_*d *_is given by:

(15) $$e_{d}=\frac{\left(a,b,c\right)}{\sqrt{a^{2}+b^{2}+c^{2}}}$$

For each simulated radial dipole, the resultant EEG at 32 electrodes, as specified by the 10–20 International electrode placement system, was calculated. Additive white Gaussian noise was then added to the noise-free data under four different signal-to-noise ratios conditions, namely 25 dB, 15 dB, 10 dB and 5 dB. To perform a Monte-Carlo analysis, for each of the 108 positions considered and for each SNR, 100 trials were simulated where the difference between trials is simply a different additive noise vector.

#### 4.2.3 Procedure

##### Current density estimate

For each scalp potential recording, the current density at each of the 755 voxels within the brain was estimated using the four different inverse solutions respectively. In order to analyze the effect of regularization on the solutions, the four methods were applied both without regularization and with regularization. For the latter case, Tikhonov regularization was used and the optimal value for the regularization parameter was found using the **L**-curve method. The MATLAB toolbox of Christian Hansen was used in this case [21].

##### Maxima

Once the current density estimates were available, the locations and normalized magnitudes of local maxima were found. A voxel was considered to hold a local maximum if the magnitude 28 of its neighbours was found to be lower than the magnitude of itself.

##### Error distance measures

The locations and magnitudes of the local maxima were used to find the deviations of estimated solution from the expected solution. Two different error measures were used compare the different inverse solutions. Error Distance measure ED1 was based on the distance between the location of the global maximum $r_{dip_{max}}$ to that of the actual position *r*_*dip *_of the simulated dipolar source.

(16) $$ED1=|r_{dip_{max}}-r_{dip}|$$

The goal of using this error measure is that for the single dipolar source scenario as considered here, this is a good measure to identify whether the largest activity corresponds to the actual simulated activity. However, in a real data scenario it is unknown as to the actual number of sources being active within the brain at a particular instant in time. For this reason, a second error measure was used which penalizes each solution for the number of resulting 'ghost' maxima. A ghost maxima is one which was not actually present in the simulated scenario. This measure, ED2, sums a weighted distance measure across the resulting number of local maxima *p *where *d*_*n *_= |$r_{dip_{n}}$ - *r*_*dip*_| and |$\hat{D}$(*n*)| is the magnitude of the local maxima $r_{dip_{n}}$ normalized to the value of the global maximum of |$\hat{D}$(*n*)|.

(17) $$ED2=\sum_{n=1}^{p}d_{n}.|\hat{D}(n)|$$

##### Statistical analysis

The error distance measures ED1 and ED2 were used as the cost functions to compare the different inverse solutions, their response in different noise conditions and the effect of regularization on the solution. Rather than analyzing the differences at each of the 108 considered dipole locations, the simulated sources were grouped into three regions made up of:

• Surface sources

• Mid-depth sources

• Deep sources

**Figure 5:**
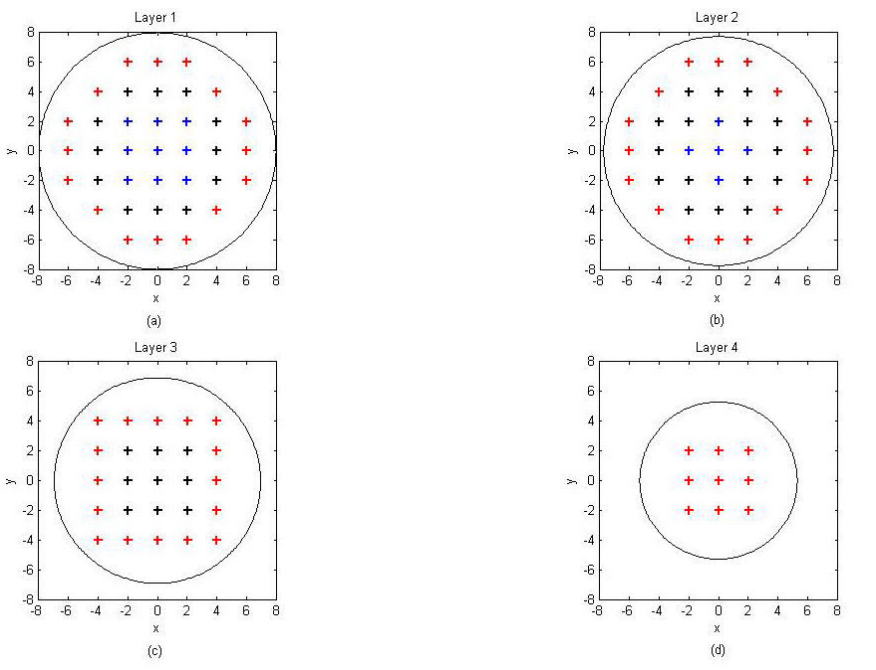
**Individual Layers in which the simulated dipoles lie**. Red crosses represent sources lying close to the surface (57 in total), black crosses represent sources lying in the middle of the spherical cortex model (37 in total) and blue crosses represent sources lying deep within the cortex (14 in total).

Figure 5 shows the individual layers in which the considered sources lie and the way in which these sources are grouped. Red crosses represent the surface sources, black crosses the mid-depth sources and blue crosses the deep sources. Layers are stacked onto each other at a distance of 2 cm.

The error distance measures for sources within each region were then averaged giving an average error value per region. These were then used to compare the different solutions. Statistical analysis of the data was then carried out through SPSS [76]. To identify whether there are significant differences between the four implemented solutions, ANOVA which is based on the following set of assumptions was used:

1. Observations within each population must be normally distributed

2. Variances between populations must be homogeneous

3. The populations represent independent random samples

Since the data being analyzed was coming from four different solutions, assumption 3 was automatically validated. If assumptions 1 and 2 were found to be true, ANOVA was then used to find whether there are significant differences between the four solutions and if such differences were found, post-hoc tests were used to identify which pairs of solutions are causing these differences. If all assumptions 1 to 3 were true, Tukey's test was used for post-hoc analysis and if the homogeneity assumption 2 was violated, Games-Howell test was used instead. If on the other hand both normality and homogeneity of variance tests failed, violating assumptions 1 and 2, then the equivalent non-parametric approach to ANOVA was used – the Kruskal-Wallis test. In this case post hoc tests were carried out using the Mann-Whitney test with Bonferroni correction [76].

#### 4.2.4 Discussion of results

Tables 4 – 5 and 6 – 7 show the averaged error distance measures, ED1 and ED2 respectively, for surface, mid-depth and deep sources, for each of the four implemented inverse algorithms.

**Table 4:** Error measure ED1 for the four inverse algorithms, without regularization, under four different noise levels: 25 dB, 15 dB, 10 dB and 5 dB. Each cell value gives the mean and standard deviation.

		**ED1**			
		**Unregularised**			

	SNR/dB	**5**	**10**	**15**	**25**
				
	Layer				

**WMN**	Surface	5.71 ± 0.49	3.75 ± 0.36	2.36 ± 0.27	1.18 ± 0.04
	
	Middle	7.21 ± 0.42	6.58 ± 0.52	5.11 ± 0.37	2.74 ± 0.18
	
	Deep	6.76 ± 0.39	6.72 ± 0.35	6.46 ± 0.39	4.98 ± 0.33

**sLORETA**	Surface	4.47 ± 0.43	2.05 ± 0.31	0.81 ± 0.13	0.04 ± 0.03
	
	Middle	6.46 ± 0.42	4.76 ± 0.43	2.12 ± 0.34	0.11 ± 0.05
	
	Deep	6.48 ± 0.37	6.01 ± 0.50	3.68 ± 0.64	0.15 ± 0.11

**LORETA**	Surface	5.49 ± 0.46	3.59 ± 0.39	2.03 ± 0.25	1.32 ± 0.02
	
	Middle	6.23 ± 0.41	5.54 ± 0.48	3.64 ± 0.44	1.14 ± 0.09
	
	Deep	5.78 ± 0.37	5.64 ± 0.37	5.30 ± 0.41	2.69 ± 0.39

**SLF**	Surface	6.38 ± 0.39	5.17 ± 0.36	3.65 ± 0.36	2.17 ± 0.14
	
	Middle	5.91 ± 0.45	5.35 ± 0.44	3.92 ± 0.41	1.92 ± 0.17
	
	Deep	5.31 ± 0.49	5.08 ± 0.43	4.46 ± 0.55	1.95 ± 0.46

**Table 5:** Error measure ED1 for the four inverse algorithms, with regularization, under four different noise levels: 25 dB, 15 dB, 10 dB and 5 dB. Each cell value gives the mean and standard deviation.

		**ED1**			
		**Regularised**			

	SNR/dB	**5**	**10**	**15**	**25**
				
	Layer				

**WMN**	Surface	3.46 ± 0.42	2.10 ± 0.28	1.34 ± 0.11	1.13 ± 0.03
	
	Middle	5.08 ± 0.50	3.94 ± 0.38	2.95 ± 0.21	2.40 ± 0.03
	
	Deep	5.91 ± 0.39	5.31 ± 0.36	4.61 ± 0.24	3.89 ± 0.15

**sLORETA**	Surface	0.99 ± 0.1	0.49 ± 0.08	0.11 ± 0.04	0.00 ± 0.00
	
	Middle	1.61 ± 0.13	0.84 ± 0.11	0.25 ± 0.07	0.00 ± 0.00
	
	Deep	1.79 ± 0.25	0.95 ± 0.16	0.39 ± 0.13	0.00 ± 0.00

**LORETA**	Surface	2.32 ± 0.08	2.18 ± 0.04	2.16 ± 0.03	2.21 ± 0.02
	
	Middle	1.51 ± 0.13	1.15 ± 0.08	0.95 ± 0.07	1.05 ± 0.06
	
	Deep	2.30 ± 0.21	1.81 ± 0.13	1.59 ± 0.11	1.53 ± 0.09

**SLF**	Surface	5.27 ± 0.30	4.50 ± 0.28	3.81 ± 0.20	2.98 ± 0.13
	
	Middle	4.53 ± 0.39	4.09 ± 0.35	3.50 ± 0.31	2.51 ± 0.15
	
	Deep	3.89 ± 0.55	3.70 ± 0.45	3.27 ± 0.48	1.73 ± 0.30

**Table 6:** Error measure ED2 for the four inverse algorithms, without regularization, under four different noise levels: 25 dB, 15 dB, 10 dB and 5 dB. Each cell value gives the mean and standard deviation.

		**ED2**			
		**Unregularised**			

	SNR/dB				
				
	Layer	**5**	**10**	**15**	**25**

**WMN**	Surface	38.49 ± 1.70	30.92 ± 1.34	19.32 ± 0.94	9.91 ± 0.40
	
	Middle	39.39 ± 2.04	37.90 ± .81	29.13 ± 1.39	18.28 ± 0.92
	
	Deep	37.65 ± 3.18	37.16 ± 3.11	31.80 ± 2.71	28.79 ± 2.21

**sLORETA**	Surface	21.35 ± 1.25	12.13 ± 0.81	4.85 ± 0.41	0.44 ± 0.05
	
	Middle	25.50 ± 1.59	21.06 ± 1.51	11.15 ± 0.83	0.83 ± 0.15
	
	Deep	24.90 ± 2.60	23.22 ± 2.31	15.57 ± 1.90	0.65 ± 0.31

**LORETA**	Surface	33.45 ± 1.16	27.10 ± 1.05	19.31 ± 0.80	8.56 ± 0.29
	
	Middle	32.17 ± 1.23	30.22 ± 1.31	25.43 ± 1.18	10.10 ± 0.56
	
	Deep	29.88 ± 1.92	29.20 ± 1.85	27.32 ± 1.71	13.95 ± 1.53

**SLF**	Surface	8.82 ± 0.66	7.19 ± 0.64	4.93 ± 0.48	2.56 ± 0.16
	
	Middle	8.17 ± 0.72	7.30 ± 0.64	5.27 ± 0.60	2.23 ± 0.23
	
	Deep	7.36 ± 0.77	6.86 ± 0.74	5.99 ± 0.83	2.56 ± 0.62

**Table 7:** Error measure ED2 for the four inverse algorithms, with regularization, under four different noise levels: 25 dB, 15 dB, 10 dB and 5 dB. Each cell value gives the mean and standard deviation.

		**ED2**			
		**Regularised**			

	SNR/dB	**5**	**10**	**15**	**25**
				
	Layer				

**WMN**	Surface	34.79 ± 1.74	25.88 ± 1.02	17.14 ± 0.71	6.91 ± 0.23
	
	Middle	35.74 ± 1.75	31.41 ± 1.87	25.6 ± 1.30	12.04 ± 0.57
	
	Deep	34.72 ± 2.50	31.14 ± 2.29	25.86 ± 2.32	16.75 ± 1.70

**sLORETA**	Surface	1.02 ± 0.10	0.49 ± 0.08	0.11 ± 0.04	0.00 ± 0.00
	
	Middle	1.62 ± 0.13	0.84 ± 0.11	0.25 ± 0.07	0.00 ± 0.00
	
	Deep	1.80 ± 0.25	0.95 ± 0.16	0.39 ± 0.13	0.00 ± 0.00

**LORETA**	Surface	6.33 ± 0.57	4.56 ± 0.25	3.97 ± 0.16	3.66 ± 0.07
	
	Middle	4.30 ± 0.46	3.16 ± 0.28	2.63 ± 0.20	1.79 ± 0.13
	
	Deep	4.20 ± 0.53	2.71 ± 0.36	2.42 ± 0.33	1.67 ± 0.14

**SLF**	Surface	6.85 ± 0.46	5.79 ± 0.44	4.82 ± 0.31	3.52 ± 0.20
	
	Middle	5.92 ± 0.65	5.38 ± 0.58	4.58 ± 0.46	3.01 ± 0.22
	
	Deep	5.25 ± 0.84	5.03 ± 0.75	4.44 ± 0.75	2.27 ± 0.42

**Figure 6:**
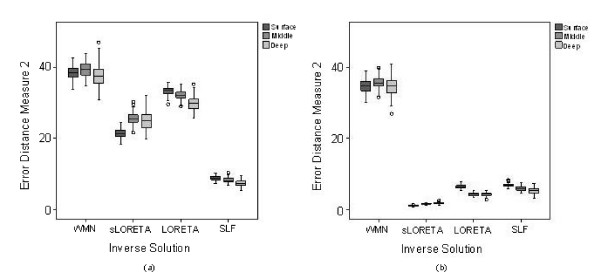
**Box-whisker diagrams**. These show the median (horizontal line within each box), the interquartile range (between the bottom and top of each box) and the range of scores (shown by the whiskers). Circles represent outliers. Plots (a) and (b) show the results for each of the four inverse solutions (horizontal axis) for error measure ED2 with a SNR of 5 dB. (a) shows the results without regularization and (b) shows the results with regularization.

Regularized sLORETA is shown to have the lowest errors both in terms of localization error (ED1) and ghost sources (ED2) (see Figure 6b). This is consistent with [32] where sLORETA was proven to have zero localization error for a single source in all noise-free simulations. Furthermore, it can be observed that the error measures and ghost maxima of sLORETA solutions can be reduced considerably when regularization is used – this improvement is particularly marked at large noise levels for ghost sources – see Figure 6 where the results for ED2 for the 5 dB scenario are displayed.

In contrast with the other algorithms studied here, SLF is an iterative method and consequently much more computationally intensive. Table 6 shows that SLF solutions without regularization have the lowest number of ghost sources; this emerges since during the iterative process voxels having a large current density are retained whereas the current density in most other voxels is set to zero. Although unregularized SLF performed better than unreg-ularized sLORETA in terms of ghost sources, unlike SLF, sLORETA benefited greatly from regularization (Table 7), reducing its ghost sources to well below those found by regularized SLF.

It is also observed that unregularized LORETA performs badly in terms of ghost sources. However, regularized LORETA is shown to reduce ghost sources considerably and to a level below regularized SLF. Furthermore, Table 5 shows that regularized LORETA solutions also have a lower localization error better than for regularized SLF. Whereas LORETA localization error and ghost sources improve greatly with regularization, WMN and SLF do not seem to benefit appreciably from regularization; in fact, SLF may be slightly worsened with regularization for low noise levels. A notable exception to the improvement of localization error of regularised LORETA occurs for surface sources with low noise levels, namely, 15 dB and 25 dB, in which case a lower error is obtained with unregularized LORETA.

Greater noise levels are expected to result in larger localization errors and ghost sources. All methods studied here have in fact followed this trend with ED1 and ED2 decreasing with higher SNRs.

For WMN and sLORETA, with and without regularization, and the unregularized LORETA, deeper sources tend to have larger localization errors and more ghost sources. Conversely, regularizing the LORETA solution resulted in higher localization errors and more ghost sources for the surface layer. Localization errors and ghost sources of SLF solutions, with or without regularization, do not appear to have a definite trend with source depth – this may be due to border artefacts involved in the SLF iterations which will affect most seriously the surface sources possibly resulting in elevated artefactual errors for the surface sources.

In summary, it can be stated that for single source localization, regularized sLORETA does indeed produce solutions with the lower localization errors and least number of ghost sources; regularization has a marked effect on these results. These statistical results also showed that regularized LORETA is the second best performing algorithm in terms of both performance measures with the exception of surface sources at low noise levels. Therefore, for single source localization, the computational cost of SLF does not yield any additional benefits over the direct methods of sLORETA and LORETA. It should also be recalled that in addition to source localization, LORETA provides source orientation estimates, which are unavailable in sLORETA solutions.

## 5 Discussion and conclusion

In EEG source analysis, the inverse problem estimates the sources within the brain giving rise to a scalp potential recording. Throughout the years various techniques have been developed to solve the inverse problem for EEG source localization and these techniques fall mainly in two categories: parametric and non parametric. The former estimates the dipole parameters of an *a priori *determined number of dipoles and the latter estimates the dipole magnitude and orientation of a number of dipoles at fixed positions distributed in the brain volume. Since in non parametric techniques the dipole location is not estimated, such techniques present a linear problem which can be solved by various methods. The non-parametric methods reviewed in this paper include MNE, LORETA, sLORETA, VARETA, S-MAP, ST-MAP, Backus-Gilbert, LAURA, Shrinking LORETA FOCUSS (SLF), SSLOFO and ALF. A series of regularization methods to approximate an ill-posed problem with a family of well-posed problems have also been discussed. On the other hand, the complexity of parametric models varies depending on the *a priori *chosen number of dipoles. Since in this case a search is made for dipole position, orientation and magnitude which appear non-linearly in the equations, parametric approaches present a non-linear problem. Parametric techniques reviewed in this paper include Beamforming techniques, BESA, subspace techniques such as MUSIC and other methods derived from it, FINES, simulated annealing and computational intelligence algorithms.

Apart from the technical details of the individual algorithms, this paper also provided a performance review of a large number of these algorithms as reported in the literature. From the non parametric techniques, LORETA was shown to give satisfactory results in most cases. However, taking into account reliable biophysical and psychological constraints as done by LAURA for example, was shown to give less localization error than solutions like LORETA. Algorithms such as SSLOFO and ST-MAP have also been developed to capture the temporal information of the individual estimated sources. In environments where there are few sources which are clustered, parametric higher resolution algorithms such as MUSIC give superior performance. Comparative analysis of parametric techniques based on signal subspace decomposition, such as MUSIC, its variants and FINES have been reported in the literature with results showing that FINES is superior in both the noise-free and noisy scenarios.

In addition to this literature review, this paper presented a Monte Carlo analysis of four widely used non parametric inverse solutions: WMN, LORETA, sLORETA and SLF. These solutions were compared at different noise levels and for simulated dipoles at different depths within the brain. Using a three-layer spherical head model, results show that for a single source, regularized sLORETA gives the best performance both in terms of localization error and ghost sources, followed by regularized LORETA. From this one could conclude that for single source localization, the computational cost of SLF does not give any additional benefits over direct methods such as LORETA and sLORETA.

The use of these techniques for EEG (and MEG) source localization in fundamental brain research and direct clinical application is today rapidly evolving. It is used not only in clinical neuroscience, i.e. neurology, psychiatry and psychopharmacology but also in cognitive neuro science research. The analyses for clinical settings differ from those used for research in the developmental neurosciences, as they are concerned largely with the identification and localization of abnormalities in the EEG [77], and the utilization of this information for neurosurgical interventions in the most severe cases [78,79].

In cognitive neuroscience such techniques have been used to localize the sources of the different frequency bands, to assess the dynamics of different mental states, such as perception, motor preparation and higher cognitive functions [80,81]. In clinical neuroscience source imaging allows the analysis of EEG changes in psychiatric and neurological patients [82-84] and is extensively used to test and characterize effects of various psychopharmacological agents [85,86].

However, the main clinical application concerns pre-surgical mapping in patients undergoing resection of tumors by allowing for better pre-surgical planning and the localization of epileptic foci as a non-invasive procedure to provide significant source of information for guiding surgical decisions. More specifically, it has been validated for the presurgical evaluation of adult patients suffering from refractory epilepsy [87-89] and in children with Landau-Kleffner syndrome [90]. Source localization is even feasible in neonates [91]. It allows the epileptogenic area to be located and comparisons to be made with clinical information, magnetic resonance imaging (MRI) anatomical data, and the results of metabolic imaging techniques. Finally, another application is in the localization of invariant quantitative EEG (QEEG) correlates of the loss and return of consciousness during anesthesia [92].

It should be noted that even though source localization has been used in many different domains, it is difficult to validate the accuracy of the results. However, when such validation was attempted on epileptic patients, even though there were some inherent limitations in localization of deep temporal structures, the results have been quite encouraging [93,94].

## Authors' contributions

RG, JM, TC, PX, MZ and VS participated in the literature search and were responsible for writing down the manuscript. TC conducted the experiments for the comparative analysis of the algorithms. KPC, TC, SGF, JM and BV participated in the design of the study and helped to draft the manuscript. All authors read and approved the final manuscript.

## Appendix

1. The minimization of *F*_*α*_(**x**) = ||**Kx **- **y**^*δ*^||^2 ^+ *α*||**x**||^2 ^leads to

$$x_{\alpha (\delta )}^{\delta }={\left(K^{*}K+\alpha I\right)}^{-1}K^{*}y^{\delta }.$$

*Proof*. Taking the derivative of *F*_*α *_and setting to zero to solve for **x **gives:   □

$$\begin{array}{c} 0=2K^{*}Kx-2K^{*}y^{\delta }-2\alpha x\\ \Rightarrow (K^{*}K+\alpha I))x=K^{*}y^{\delta }\\ \Rightarrow x_{\alpha (\delta )}^{\delta }={\left(K^{*}K+\alpha I\right)}^{-1}K^{*}y^{\delta }. \end{array}$$

2. The inverse operators (K*K + *α***I**)^-1^K* and K*(KK* + *α***I**)^-1 ^are equal.

*Proof*. Assume **K **is a *N *× *p *linear operator. Then   □

$$\begin{array}{c} K^{*}KK^{*}+\alpha K^{*}I_{N}=K^{*}KK^{*}+\alpha I_{p}K^{*}\\ \Rightarrow (K^{*}KK^{*}+\alpha I_{N})=(K^{*}K+\alpha I_{p})K^{*}\\ \Rightarrow {\left(K^{*}K+\alpha I_{p}\right)}^{-1}K^{*}(KK^{*}+\alpha I_{N}){\left(KK^{*}+\alpha I_{N}\right)}^{-1}=\\ {\left(K^{*}K+\alpha I_{p}\right)}^{-1}(K^{*}K+\alpha I_{p})K^{*}{\left(KK^{*}+\alpha I_{N}\right)}^{-1}\\ \Rightarrow {\left(K^{*}K+\alpha I_{p}\right)}^{-1}K^{*}I_{N}=I_{p}K^{*}{\left(KK^{*}+\alpha I_{N}\right)}^{-1}\\ \Rightarrow {\left(K^{*}K+\alpha I_{p}\right)}^{-1}K^{*}=K^{*}{\left(KK^{*}+\alpha I_{N}\right)}^{-1}. \end{array}$$

3. The solution of the LCMV problem

$$\begin{array}{ccc} \underset{W^{T}}{\min}Tr\{W^{T}C_{m}W\} & \text{subject to} & W^{T}G(r_{dip})=I \end{array}$$

is $W(r_{dip})={\left[G{\left(r_{dip}\right)}^{T}C_{m}^{-1}G(r_{dip})\right]}^{-1}G{\left(r_{dip}\right)}^{T}C_{m}^{-1}$.

*Proof*. Let 2**L **be a 3 × 3 matrix of Lagrange multipliers. The cost function is added with the inner product of the Lagrange multipliers and the constraint to obtain the Lagrangian *L*(**W**, **L**):

*L*(**W**, **L**) = *Tr*{**W**^*T*^**C**_**m**_**W **+ (**W**^*T*^**G **- **I**)2**L**}.

Noting that *Tr*{**A**} = *Tr*{**A**^*T*^} for any square matrix **A**, the above equation can be rewritten as:

$$\begin{array}{c} L(W,L)=Tr\{W^{T}C_{m}W+(W^{T}G-I)L+L^{T}(G^{T}W-I)\}\\ =Tr\{(W^{T}+L^{T}G^{T}C_{m}^{-1})C_{m}(W+C_{m}^{-1}GL)-L-L^{T}-L^{T}G^{T}C_{m}^{-1}GL\}. \end{array}$$

Only the first term in the brackets is a function of **W**. The matrix **C**_**m **_is positive definite so the minimum of *L*(**W**, **L**) is attained by setting the first term to zero, that is:

(18) $$W=-C_{m}^{-1}GL.$$

The Lagrange multiplier matrix **L **is now obtained by substituting **W **in the constraint **W**^*T*^**G **= **I **to obtain:

$$-L^{T}G^{T}C_{m}^{-1}G=I$$

or

(19) $$L^{T}=-{\left(G^{T}C_{m}^{-1}G\right)}^{-1}.$$

Substituting Equation (19) in Equation (18) yields the solution:   □

$$W(r_{dip})={\left[G{\left(r_{dip}\right)}^{T}C_{m}^{-1}G(r_{dip})\right]}^{-1}G{\left(r_{dip}\right)}^{T}C_{m}^{-1}.$$
